# The *Drosophila* pseudokinase Tribbles translocates to the fat body membrane in response to fasting to modulate insulin sensitivity

**DOI:** 10.1242/dev.204493

**Published:** 2025-04-28

**Authors:** Zachary Fischer, Christopher Nauman, Shima Shayestehpour, Laramie Pence, Samuel Bouyain, Xiaolan Yao, Leonard L. Dobens

**Affiliations:** Division of Biology and Biomedical Engineering, School of Science and Engineering, University of Missouri-Kansas City, Kansas City, MO 64110, USA

**Keywords:** Trib protein family, Pseudokinase, Growth, Insulin signaling

## Abstract

The *Drosophila* pseudokinase Tribbles (Trbl) shares conserved functions with human TRIB3 to bind and inhibit Akt phosphorylation-activation by the Insulin Receptor (InR) to reduce insulin responses; consistent with this, increased levels of human TRIB3 are linked to type 2 diabetes. Here, we show that in fat body cells of well-fed *Drosophila* larvae, Trbl expression is low and predominantly in the nucleus while fasting or genetic reduction of insulin signaling resulted in increased Trbl expression and Trbl protein translocation to the plasma membrane. An E/G mutation in the Trbl pseudokinase kinase activation loop dominantly interfered with Trbl function leading to increased Akt activity, increased stability of Trbl substrates, including Trbl itself, and aberrant redistribution of Trbl multimers to the membrane. Several strategies designed to increase Akt activity were sufficient to translocate Trbl to the membrane, consistent with the notion that subcellular trafficking of Trbl to the fat body cell membrane acts a rheostat to reduce the strength of Akt-mediated insulin responses, counter to the InR, which has been shown to redistribute away from the membrane to modulate insulin signaling.

## Teaser

**Summary:** In response to dietary stress, the *Drosophila* pseudokinase Tribbles (Trib) translocates to the membrane to block Akt activation and mutation of the conserved pseudokinase activation loop dominantly disrupts translocation.

## INTRODUCTION

Members of the Tribbles (Trib) family of proteins act as adaptor proteins to bind and inhibit key targets that regulate cell proliferation, tissue differentiation and insulin growth during normal development and in response to environmental signals (reviewed by Dobens et al., 2021; Eyers et al., 2017; Richmond and Keeshan, 2020; Jadhav and Bauer, 2019; Zhang et al., 2021; Hernandez-Resendiz and Burkhardt, 2024; Lei et al., 2024). Trib family members share a structure similar to catalytically active kinases, with a conserved central region connected to N- and C-lobes (Kornev and Taylor, 2015; Shrestha et al., 2020). The arrangement of motifs in the central region of human TRIB1 precludes ATP binding, and thus these proteins are deemed pseudokinases, although low kinase activity has been reported for TRIB2 (Bailey et al., 2015). Substrate binding results in an allosteric shape change in the central pseudokinase domain's activation loop, which swings from an extended ‘out’ conformation to an ‘in’ configuration, leading to release of an intramolecularly bound C-terminal tail (CTT), which recruits an E3 ligase via a COP1-binding site in the tail domain to catalyze ubiquitin-mediated turnover of substrate (Jamieson et al., 2018, 2022; Murphy et al., 2015; reviewed in Velasco and Link, 2023). TRIB1, TRIB2 and *Drosophila* Trbl bind and degrade conserved targets including CDC25 phosphatase to block cell division, and C/EBP to regulate cell differentiation (Grosshans and Wieschaus, 2000; Mata et al., 2000; Rørth et al., 2000; Seher and Leptin, 2000; Keeshan et al., 2006; Selim et al., 2007; Masoner et al., 2013; Murphy et al., 2015; Kim et al., 2016; Liang et al., 2016; McEwan et al., 2016; Otsuki and Brand, 2018; Liu et al., 2020). In *Drosophila*, Tribbles mediates Hippo pathway-regulated tumor growth (Gerlach et al., 2019), and mammalian Trib1 and Trib2 act as either oncogenes or tumor suppressors depending on the tissue context (Gilby et al., 2010; Wang et al., 2013a; Liang et al., 2013; Miyajima et al., 2015; Li et al., 2020; Salomé et al., 2018; Hong et al., 2019; Qu et al., 2019; Singh et al., 2024; Fang et al., 2021; Stefanovska et al., 2021; reviewed by Mayoral-Varo et al., 2021; Ferreira et al., 2021; McMillan et al., 2021; Arif et al., 2023).

The discovery that the Trib3 isoform in mice inhibits insulin responses by binding Akt kinase to prevent its phosphorylation-activation by the insulin receptor (Insr) led to extensive studies on the role of Trib proteins in insulin responses and metabolic disease (Du et al., 2003). In the mouse model, Trib3 levels increase following either over-feeding, starvation or exercise to increase insulin insensitivity (Bi et al., 2008; Schwarzer et al., 2006; Marinho et al., 2012; Liu et al., 2012; Örd et al., 2014; Canciglieri et al., 2018; de Souza Cordeiro et al., 2019; Popovic et al., 2023; Lima et al., 2009; Matos et al., 2010; reviewed by Lu et al., 2024). In humans, *TRIB3* variants with increased Akt binding strength have been associated with diabetes and cardiovascular disease (De Cosmo et al., 2007; Prudente et al., 2005, 2009, 2010, 2012; Liu et al., 2010; Weismann et al., 2011; Fischer et al., 2017; Lee et al., 2022; Salazar et al., 2015; reviewed by Prudente and Trischitta, 2015). In *Drosophila*, Trbl inhibits Akt kinase to block phosphorylation and mute insulin responses and the effects of administering a high-fat diet (HFD) can be reversed by Trbl RNAi knockdown, indicating that Trbl is a conserved mediator of dietary stress phenotypes (Das et al., 2014; Hong et al., 2019).

Here, we use the larval fat body model to show that *Drosophila* Trbl protein is predominantly nuclear in well-fed animals and that upon fasting *trbl* gene expression levels increase and protein accumulates at the cell membrane to block Akt activation. Mutational analysis of the conserved pseudokinase domain identified a residue in the activation loop that dominantly blocks Trbl instability and Akt inactivation leading to mislocalization of Trbl protein. Together with previous work showing that the *Drosophila* insulin receptor complex localizes to the membrane in response to activity (Kim et al., 2018), these data suggest that environmental cues modulate insulin sensitivity in the fly fat body by regulating membrane association of key components of the insulin signaling pathway.

## RESULTS

### Trbl expression increases in response to fasting and selectively binds activated Akt at the fat body cell membrane

**Fig. 1.**
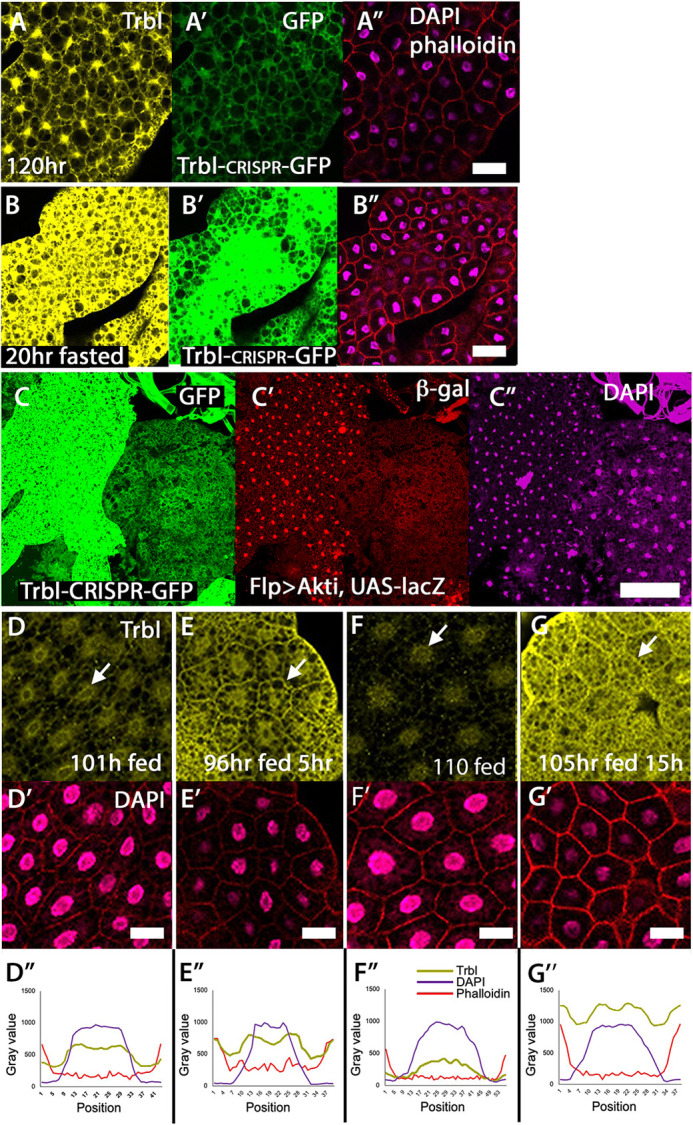
***trbl* gene expression increases and Trbl protein accumulates at the larval fat body cell membrane in response to fasting.** (A-A″) Fat body expression of Trbl protein (yellow; A) and a Trbl-GFP reporter gene (green; A′) are low at 120 h AED (after egg deposition) in fed larvae (DAPI and phalloidin staining in purple and red, respectively; A″). Genotype: Trbl-GFP/TM3Sb. (B-B″) Expression of Trbl protein (yellow; B) and a Trbl-GFP reporter gene (green; B′) increased at 111 h in larvae that were fasted for 15 h (DAPI and phalloidin staining in purple and red, respectively; B″). Genotype: Trbl-GFP/TM3Sb. (C-C″) At 101 h AED, Trbl-GFP reporter gene levels increased significantly (green; C) in Flp-out clones expressing a UAS-controlled RNAi to Akt marked by UAS-*lacZ* expression (red; C′). DAPI staining is shown in C″ (purple). Genotype: hsFLP; AyGAL4; UAS-Akt^i^/Trbl-GFP. (D-D″) At 101 h AED, endogenous Trbl protein localized to the nucleus (yellow; D, arrow) with lower levels of protein accumulation at the cell membrane and in the cytoplasm in fat body cells of fed larvae. Genotype: Canton S. DAPI and phalloidin staining are shown in purple and red, respectively, in D-G). (E-E″) At 101 h AED fasted for 5 h, Trbl protein levels (yellow; E) increased and Trbl protein localized more strongly to the cell membrane (arrow) with corresponding lower nuclear levels. Genotype: Canton S. (F-F″) At 111 h AED, endogenous Trbl protein accumulated in the nucleus (arrow) with lower protein levels at the cell membrane and in the cytoplasm. Genotype: Canton S. (G-G″) At 111 h AED fasted for 15 h, Trbl protein levels increased compared to well-fed animals and Trbl protein localized more strongly to the cell membrane (arrow) with corresponding lower nuclear levels. Genotype: Canton S. (D″-G″) Line graphs of plot profiles showing average distribution of fluorescence intensity of DAPI, phalloidin and anti-Trbl across individual cells from the respective tissues shown above (*n*=90 cells). Genotype: Canton S. Scale bars: 50 µm.

In mammalian models, Trib3 expression increases in response to starvation (Lima et al., 2009; Matos et al., 2010), so to test whether *Drosophila* Trbl expression is sensitive to dietary stress, we examined expression of a Trbl-GFP Crispr knock-in reporter transgene (construction described in Materials and Methods) in the larval fat body following fasting (Fig. 1, Fig. S1). Insulin-sensitive fat body cells arise from embryonic precursors to form two parallel epithelial cell sheets running the length of the body cavity; throughout larval stages, these adipose cells grow by both nuclear endoreduplication and lipid uptake (reviewed by Musselman and Kühnlein, 2018; Tonk-Rügen et al., 2022; Chatterjee and Perrimon, 2021). In wild-type (WT) fat body tissue at 120 h of development, we observed low levels of expression of Trbl-GFP (Fig. 1A), while in larvae fasted for 20 h Trbl reporter gene expression was noticeably increased (Fig. 1B), and this increase was reflected in increased Trbl protein levels detected with specific antisera (compare Fig. 1A′ and B′). To test whether reduced insulin signaling is responsible for the increased Trbl-GFP expression in response to dietary stress, we produced Flp-out marked fat body cell clones expressing an RNAi to Akt (Perkins et al., 2015). As shown in Fig. 1C-C″, Akt RNAi clones marked with UAS-*lacZ* (Fig. 1C′, red) resulted in a strong increase in Trbl-GFP expression (Fig. 1C), along with a noticeable reduction in cell and nuclear size (Fig. 1C″) in these cells in which insulin responses have been genetically blocked. We conclude that fat body cells respond to dietary stress and reduced insulin responses by increasing *trbl* gene expression and that this occurs in a cell-autonomous manner.

In well-fed larvae collected at 101 h or 111 h, the Trbl antiserum detected protein primarily in the cell nucleus and its periphery (Fig. 1D,F, arrows), with low levels in the cytoplasm and at the fat cell membrane. In well-fed animals, Trbl protein accumulated more strongly at the cell membrane of a subset of posterior fat body cells (Fig. S1E), suggesting that the subcellular protein distribution may be regulated in some subdomains of the fat body organ. To examine whether dietary stress modulates Trbl localization, we fasted larvae for either 5 h (Fig. 1E, at 101 h) or 15 h (Fig. 1G, at 111 h), and observed increased Trbl protein levels at the membrane and a corresponding reduction of nuclear levels. We observed stronger localization of Trbl to the membrane with longer fasting (Fig. 1E,G, arrows). These changes in Trbl protein distribution in response to fasting were documented by cross-sectional scan measurements of Trbl fluorescence distribution compared to fluorescence of rhodamine-conjugated phalloidin (detecting actin at the cell membrane) and DAPI (detecting DNA in the nucleus) in at least 60 cells (summarized in Fig. 1D″-G″). A pan-Akt antiserum detected Akt localization in the nucleus and cell membrane of well-fed animals, and Akt levels and localization did not change greatly in fasted animals (compare Fig. S1C,D to Fig. S1F,G).

### Trbl pseudokinase activation loop regulates distribution to the membrane

**Fig. 2.**
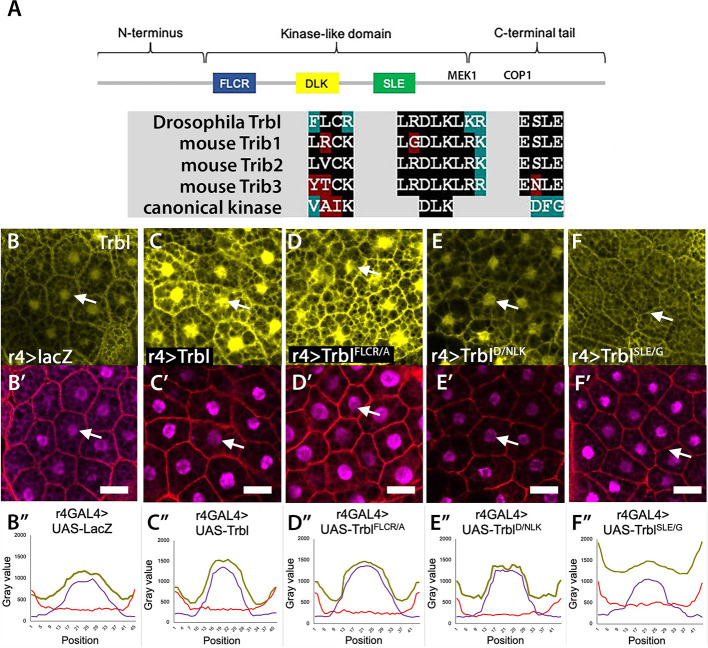
**The activation loop mutation Trbl^SLE/G^ strongly accumulates at the membrane in well-fed animals.** (A) Top: Map of Trbl protein indicating the position of conserved motifs in the kinase-like core and C-terminal tail. Bottom: Sequence alignment of motifs in *Drosophila* Trbl with mouse Trib1, Trib2 and Trib3; noted are sequence divergence of Trib pseudokinases from canonical kinases motifs. (B-F″) Subcellular distribution of endogenous Trbl and Trbl transgenes bearing kinase core mutations (yellow; B-F) with phalloidin (red) and DAPI (magenta) overlay (B′-F′) and line graphs (B″-F″) depicting the average fluorescence intensity of Trbl, DAPI and phalloidin (yellow, magenta and red, respectively; *n*=90 cells). (B,B′) Endogenous Trbl accumulates in nucleus (arrow), with lower levels in cytoplasm and at the membrane. Genotype: Canton S. (C,C′) R4-GAL4 expression of Flag-Trbl results in a distribution of Trbl protein comparable to endogenous Trbl (B). Genotype: R4GAL4>UAS-Flag-Trbl. (D,D′) R4-GAL4 expression of Flag-TrblFLCR/A distribution is similar to WT Trbl. Genotype: R4GAL4>UAS-Flag-TrblFLCR/A. (E,E′) R4-GAL4 expression of Flag-TrblD/NLK distribution is similar to WT Trbl. Genotype: R4GAL4>UAS-Flag-TrblD/NLK. (F,F′) R4-GAL4 expression of Flag-TrblSLE/G results in low levels in the nucleus with strongly increased accumulation at the cell membrane (arrow). Genotype: R4GAL4>UAS-Flag-TrblSLE/G. Scale bars: 50 µm.

These data support the notion that, in response to fasting, Trbl translocation to the membrane reduces Akt activity. To identify the motifs that might mediate this effect, we produced site-specific mutations in conserved domains of Trbl and tested for changes in subcellular distribution in well-fed animals. Using a UAS-regulated Flag-tagged Trbl protein (Fig. 2A), we produced a variety of mutations, including in (1) the DLK motif, which is conserved in all Trib family members and conserved with kinases (aspartate with an asparagine, Trbl^D/NLK^); (2) the divergent FLCR motif, which corresponds in position to the VAIK motif required for ATP coordination in bona fide kinases (an arginine to alanine, TrblFLCR/A; Carrera et al., 1993); and (3) the SLE motif, which is conserved in the proposed activation loop of Trib family members but diverges from the DFG motif in the corresponding position of the loop in bona fide kinases (a glutamate to glycine, TrblSLE/G; Taylor et al., 2013). A complete list of mutations in the N-lobe, the pseudokinase central domain and the C-terminal tail tested in transgenic flies for functions is given in Table S1.

Fat body expression of UAS-Flag-Trbl^FLCR/A^ (Fig. 2D) or Flag-Trbl^D/NLK^ (Fig. 2E) resulted in a protein distribution similar to WT Trbl (Fig. 2B,C) in well-fed animals (detected by Trbl antiserum in Fig. 2 and by Flag staining in parallel presented in Fig. S2A′-F′). In contrast, for the mutation Flag-Trbl^SLE/G^ we observed aberrant accumulation of Trbl and Flag staining at the cell membrane with corresponding reduced nuclear and cytoplasmic levels (Fig. 2F, arrow; Fig. S2B). This dramatic change in distribution of Trbl^SLE/G^ is evident in cross-section scans of Trbl fluorescence relative to fluorescence from DAPI staining of nuclei and phalloidin staining of actin at the cell membrane (Fig. 2B″-F″).

To validate that the mislocalization of Flag-Trbl^SLE/G^ to the membrane is due to a single glycine replacement in the conserved ESLE activation loop, we tested two independent UAS-Flag-Trbl^SLE/G^ insertions on both the second and third chromosomes and sequenced completely both the original vector pUAST-Flag-Trbl^SLE/G^ and genomic DNA from the corresponding transgenic animals (Fig. S10). Further, we used a C-terminal HA tag (UAS-Trbl-3xHA; Bischof et al., 2013), which shows HA accumulation in the cytoplasm, cell membrane and nucleus similar to endogenous Trbl (Fig. 4G-G″) to introduce the SLE/G mutation. As shown in Fig. 4H-H″, fat body expression of this independently derived UAS-Trbl^SLE/G^-HA exhibited aberrant membrane accumulation identical to that of Flag-Trbl^SLE/G^. Use of the fat body-specific Pumpless-GAL4 driver to express Trbl^SLE/G^ also resulted in high levels of Trbl accumulation at the membrane compared to WT Trbl (Fig. S4H).

### The Trbl activation loop modulates the strength of Trbl–Akt interactions

**Fig. 3.**
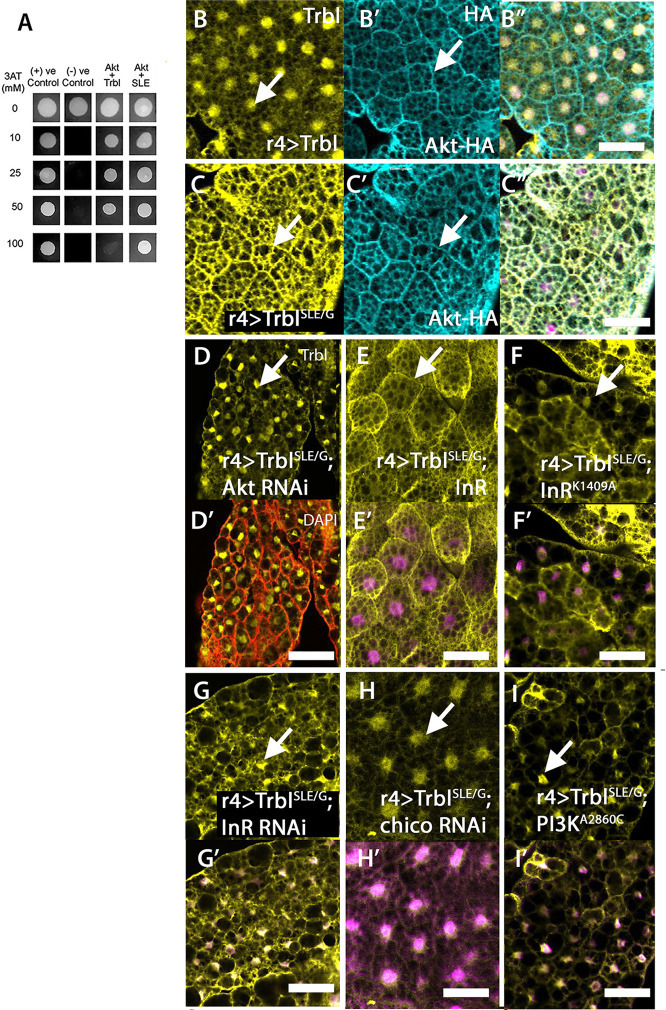
**Membrane association of Trbl^SLE/G^ depends on the level of Akt activation.** (A) Yeast two-hybrid analysis comparing Akt binding strength to Trbl and TrblSLE/G. Akt-Trbl^SLE/G^ showed growth on media up to 100 mM 3AT while Akt-Trbl showed growth up to 50 mM 3AT. (B-B″) R4-GAL4 co-expression of Trbl and HA-tagged Akt revealed that Trbl protein expression is stronger in the nucleus (B, arrow) and accumulates at lower levels in the cytoplasm and cell membrane while HA-Akt is strong at the cell membrane (B′, arrow). DAPI overlay is shown in B″. Genotype: R4GAL4>UAS-Flag-Trbl, UAS-HA-dAkt. (C-C″) R4-GAL4 co-expression of Trbl^SLE/G^ and HA-tagged Akt showed that Trbl protein accumulates at the cell membrane (C, arrow) while HA-Akt remains unchanged in localization and levels at the cell membrane (C′, arrow). DAPI overlay is shown in C″. Genotype: R4GAL4>UAS-Flag-Trbl^SLE/G^, UAS-HA-dAkt. (D,D′) R4-GAL4 co-expression of a UAS-regulated RNAi transgene to Akt and Flag-Trbl^SLE/G^ resulted in reduced accumulation of Trbl at the membrane (compared to Flag-Trbl^SLE/G^ alone; C) and the appearance of ectopic nuclear Trbl (D, arrow). Phalloidin overlay is shown in D′. Genotype: R4GAL4>UAS-Flag-Trbl^SLE/G^, UAS-dAkt RNAi. (E,E′) R4-GAL4 co-expression of UAS-InR (WT insulin receptor) and Flag-Trbl^SLE/G^ resulted in strong accumulation of Trbl at the membrane (similar to Flag-Trbl^SLE/G^ alone; C). DAPI overlay is shown in E′. Genotype: R4-GAL4>UAS-InR, UAS-Flag-Trbl^SLE/G^. (F,F′) R4-GAL4 co-expression of a UAS-regulated, dominant-negative version of the insulin receptor (InRK1409A) and Flag-Trbl^SLE/G^ resulted in reduced accumulation of Trbl at the membrane (compared to Flag-Trbl^SLE/G^ alone) and the appearance of nuclear Trbl (F, arrow). DAPI overlay is shown in F′. Genotype: R4-GAL4>UAS-InRK1409A, UAS-Flag-Trbl^SLE/G^. (G,G′) R4-GAL4 co-expression of an RNAi transgene to InR and Flag-Trbl^SLE/G^ resulted in reduced levels of Trbl accumulation at the membrane (compared to Flag-Trbl^SLE/G^ alone) and variable nuclear accumulation (G, arrow). DAPI overlay is shown in G′. Genotype: R4-GAL4>UAS-InR RNAI, UAS-Flag-Trbl^SLE/G^. (H,H′) R4-GAL4 co-expression of an RNAi transgene to *chico*, which encodes the IRS (insulin receptor substrate) and Flag-Trbl^SLE/G^ resulted in reduced levels of Trbl accumulation at the membrane (compared to Flag-Trbl^SLE/G^ alone) and increased nuclear accumulation (H, arrow). DAPI overlay is shown in H′. Genotype: R4-GAL4>UAS-*chico* RNAI, UAS-Flag-Trbl^SLE/G^. (I,I′) R4-GAL4 co-expression of UAS-regulated, dominant-negative version of PI3K (PI3KA2860C) and Flag-Trbl^SLE/G^ resulted in both reduced accumulation of Trbl at the membrane (compared to Flag-Trbl^SLE/G^ alone) and the appearance of nuclear Trbl accumulation (arrow). DAPI overlay is shown in I′. Genotype: R4-GAL4 UAS-PI3KA2860C and Flag-Trbl^SLE/G^. Scale bars: 50 µm.

The membrane association of Flag-Trbl^SLE/G^ in well-fed animals suggested that this activation loop mutation may increase Trbl affinity for activated Akt. To test this, we took *in vitro* and *in vivo* approaches. First, we cloned the Akt open reading frame (ORF) into a pDEST22 prey vector co-transfected with a bait vector for either WT Trbl (Masoner et al., 2013) or Trbl^SLE/G^. To measure the strength of Trbl–Akt interactions, we compared colony growth of co-transformants in media containing increasing concentrations of 3-AT, a competitive inhibitor of the *His3* gene (Causier and Davies, 2002). From this approach, we observed that yeast transformed with Trbl^SLE/G^ in the bait and Akt in the prey grew on higher concentrations of 3-AT than the Trbl bait-Akt prey co-transformants (Fig. 3A).

We showed previously that WT Trbl has no effect on endogenous Akt levels (Fischer et al., 2017) and, as shown in Fig. 3B,C, co-expression of Flag-Trbl or Flag-Trbl^SLE/G^ resulted in similar levels and localization of an HA-tagged version of Akt (compare Fig. 3B′ and C′). To test whether the aberrant membrane localization of Trbl^SLE/G^ is affected by changes in Akt activation, we co-expressed Trbl^SLE/G^ with several transgenes expected to reduce Akt activity. Co-expression of Trbl^SLE/G^ and a double-stranded RNA directed towards Akt (UAS-Akt^i^) resulted in reduced Trbl^SLE/G^ accumulation at the membrane in most fat body cells, with a corresponding increase in nuclear accumulation (Fig. 3D, arrow). Although co-expression of WT InR did not modify the localization of Flag-Trbl^SLE/G^ with the membrane (Fig. 3E, arrow), co-expression of either a dominant-negative InR (K1409A; Fig. 3F) or an RNAi directed against InR (Fig. 3G) reduced Flag-Trbl^SLE/G^ accumulation at the membrane and increased nuclear staining. Similarly, expression of transgenes predicted to reduce levels of activated Akt also reduced Trbl^SLE/G^ staining at the membrane, including (1) RNAi to the Chico insulin receptor substrate (IRS) homolog (Fig. 3H), (2) RNAi to PI3K (Fig. S3I) and (3) a UAS-regulated PI3K^A2860C^ dominant-negative allele (Fig. 3I, Fig. S3H). In addition, downstream targets known to feed back and modulate Akt activation also reduced Trbl^SLE/G^ membrane accumulation and increased detectable nuclear levels of Trbl^SLE/G^ staining, including (1) misexpression of an activated form of S6 kinase S6KSTDE shown to reduce InR levels (S6KSTDE; Fig. S3J; Barcelo and Stewart, 2002; Kockel et al., 2010) and (2) Tsc1/Tsc2 (Gig) co-expression. (Fig. S3K; Potter et al., 2001). We conclude that Trbl^SLE/G^ membrane association is dependent on activated Akt.

### The Trbl activation loop is required for Trbl instability

**Fig. 4.**
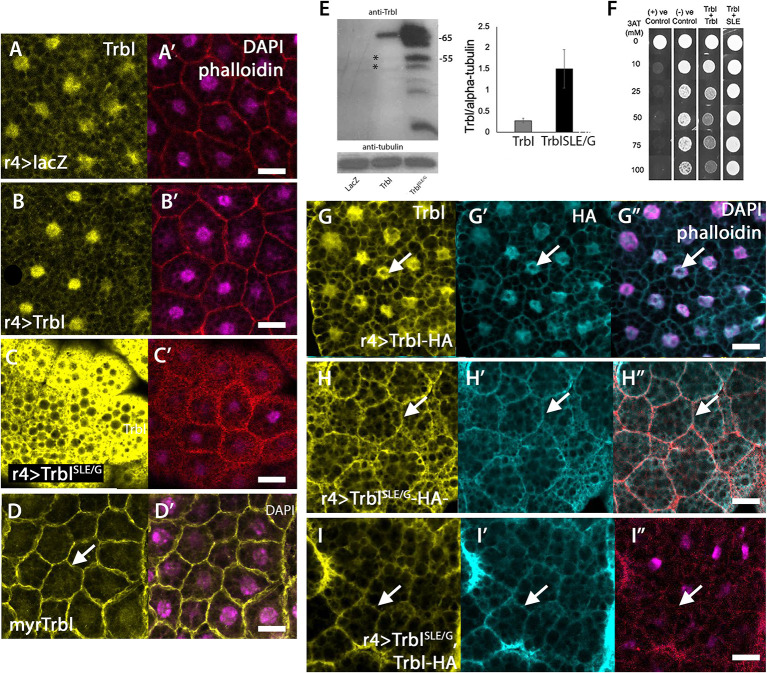
**Trbl^SLE/G^ stabilizes Trbl complexes at the fat body membrane.** (A-C′) Comparison of Trbl levels in fat body expressing UAS-*lacZ* (A), UAS-Flag-Trbl (B) and UAS-Flag-Trbl^SLE/G^ stained in parallel with Trbl antisera. Much higher levels of the Trbl^SLE/G^ protein were detected (C) compared to WT Trbl (B), which had levels comparable to endogenous levels (A). DAPI and phalloidin staining is shown in A′-C′. (D,D′) R4-GAL4 co-expression of HA-myr-Trbl and UAS-Trbl resulted in strong membrane localization of both myr-Trbl and endogenous Trbl, which is absent from the nucleus (D, arrow). DAPI overlay is shown in D′. Genotype: R4-GAL4>HA-myr-Trbl. (E) Western blot analysis comparing Trbl levels in fat body lysate from animals expressing Trbl and Flag-Trbl^SLE/G^ (left) shows higher levels of Flag-Trbl^SLE/G^ protein levels compared to WT Flag-Trbl expressed under identical conditions from the same attB landing site and sixfold increased stability of endogenous Trbl and breakdown products detectable by antisera to a C-terminal epitope (right; results are from scans of three western blots, averaged). Error bars represent s.d. *n*=3 groups of 40 age-matched larvae. (F) Yeast two-hybrid analysis comparing the binding strength of Trbl-Trbl to Trbl^SLE/G^-Trbl showed that the stronger Flag-Trbl^SLE/G^ prey had growth up to 100 mM 3AT while WT Trbl growth was reduced at this level of 3AT. (G-G″) R4-GAL4 expression of C-terminally tagged Trbl-HA showing that the fat body cell distribution of HA staining is stronger in the nucleus (arrow) and weaker in the cell membrane and cytoplasm. DAPI and phalloidin staining are shown in G″. Genotype: R4-GAL4>UAS-TrblHA. (H-H″) R4-GAL4 expression of C-terminally tagged Trbl^SLE/G^-HA showing that the fat body cell distribution of HA is strong at the cell membrane (arrow), and variable in the nucleus. DAPI and phalloidin staining are shown in H″. Genotype: R4-GAL4>Trbl^SLE/G^-HA. (I-I″) R4-GAL4 driving co-expression of Flag-Trbl^SLE/G^ and Trbl-HA detects HA strongly at the cell membrane (arrow). DAPI/phalloidin overlay shown in I″. Genotype: R4-GAL4>Flag-Trbl^SLE/G^, Trbl-HA. Scale bars: 50 µm.

R4-GAL4 expression of WT UAS-Flag-Trbl (Fig. 4B) led to Trbl protein levels that were slightly higher than endogenous Trbl levels in the fat body (Fig. 4A), as detected by whole-mount immunofluorescence. In contrast, expression of UAS-Flag-Trbl^SLE/G^ resulted in dramatically higher levels of detectable Trbl when stained in parallel (Fig. 4C) despite both transgenes being (1) inserted at the same attB landing site on the second chromosome and (2) under the control of the same R4-GAL4 driver. To measure Trbl protein levels in these tissues, we prepared extracts from hand-dissected fat body expressing each transgene and used western blots probed with specific antisera designed to recognize an epitope in the C-tail. As shown in Fig. 4E, we detected a ∼65 kDa band corresponding to the predicted sizes of the Flag fusion protein in both Flag-Trbl and Flag-Trbl^SLE/G^ extracts; however, this band was present at sixfold higher levels in Flag-Trbl^SLE/G^ extracts (Fig. 4E, right). Also, from tissue extracts expressing Flag-Trbl^SLE/G^ we noticed the appearance of a strong band at ∼55 kDa that corresponds in size to endogenous Trbl, which we can detect in the Trbl- and control *lacZ*-expressing lanes in overexposed blots (Fig. 4E; Fig. S11). These data indicate that the Trbl^SLE/G^ mutation dominantly reduces the instability of endogenous Trbl and several of its smaller isoforms (or breakdown products).

In mammals, Trib2 dimers have been documented (Jamieson et al., 2022) and we have previously demonstrated that Trbl–Trbl interactions occur in *Drosophila* (Masoner et al., 2013), so we tested the notion that the SLE/G mutation stabilizes Trbl hetero-multimers in several ways. First, we used the yeast two-hybrid approach and engineered the SLE/G mutation in the bait vector fused in-frame with the coding sequence for a yeast DNA-binding domain and tested the effect of the activation loop mutation on Trbl–Trbl interactions using a prey vector consisting of the WT Trbl ORF fused in-frame with the coding sequence for a yeast activation domain. As shown in Fig. 4F, Trbl^SLE/G^-Trbl bait-prey co-transformants grew on higher concentrations of 3-AT than the Trbl-Trbl bait-prey co-transformants. This outcome is consistent with increased strength of Trbl^SLE/G^-Trbl heterodimers relative to Trbl-Trbl dimers. To test the significance of this *in vivo*, we expressed Flag-Trbl^SLE/G^ together with an HA-tagged version of WT Trbl (Trbl-HA; Bischof et al., 2013). As noted above, expression of UAS-Trbl-HA alone showed accumulation identical to that of endogenous Trbl: stronger in the fat body cell nucleus (Fig. 4G-G″, arrow), weaker in the cytoplasm and weakly detectable at the cell membrane. Co-expression of Flag-Trbl^SLE/G^ with this HA-Trbl resulted in HA staining strongly at the membrane (Fig. 4I-I″, arrow), with a concomitant reduction in nuclear HA staining (compare Fig. 4G and I, G′ and I′). Together, these data suggest that the Trbl^SLE/G^ activation loop mutation acts in trans to bind endogenous Trbl, mislocalize it to the membrane and aberrantly enhance its stability. In support of the ability of Trbl^SLE/G^ to bind and mislocalize endogenous Trbl, a myristoylation tag added to the N terminus of WT Trbl is sufficient to localize endogenous Trbl from the nucleus to the membrane (Fig. 4D; corresponding fat body organs shown in Fig. S4).

### A mutation in the Trbl activation loop exerts dominant-negative effects on Trbl function

**Fig. 5.**
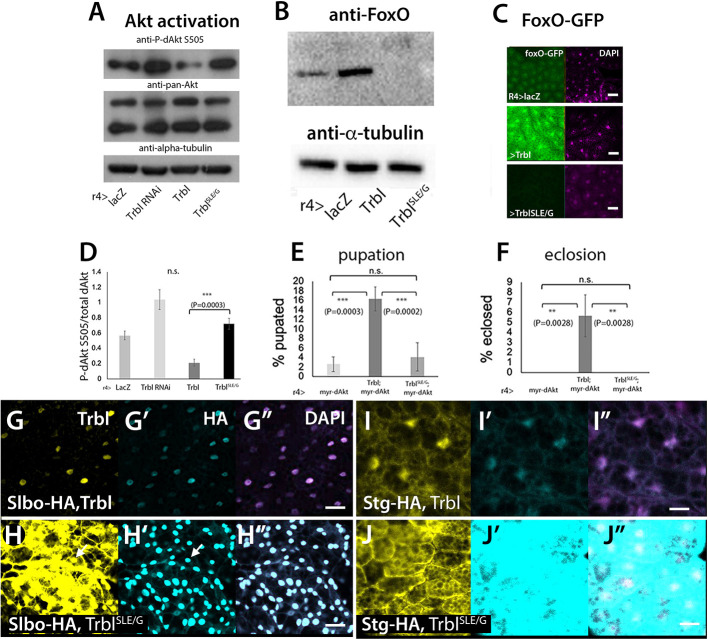
**Trbl^SLE/G^ has dominant-negative effects on Akt activation and on Trbl target instability.** (A) Western blot probed with phospho-S505-dAkt antisera (top), pan-dAkt sera (middle) and anti-tubulin (bottom) from purified lysates derived from hand-dissected larval fat body expressing *lacZ* as a control, Trbl RNAi, WT Flag-Trbl and Flag-Trbl^SLE/G^ (lanes 1-4, respectively). As shown before, Trbl RNAi expression increased P-Akt levels and Flag-Trbl expression reduced P-Akt levels compared to controls. In contrast, Flag-Trbl^SLE/G^ expression resulted in increased P-Akt compared to the *lacZ* control. Total levels of Akt as detected by pan-Akt revealed no change in levels of this target under these treatments. (B) Western blot probed with sera to detect FoxO and tubulin in lysates from hand-dissected larval fat body expressing *lacZ* as a control, WT Flag-Trbl and Flag-Trbl^SLE/G^. While Flag-Trbl expression increased FoxO levels compared to controls, Flag-Trbl^SLE/G^ expression decreased FoxO levels. (C) FoxO-GFP levels are low in the nucleus of fat body dissected from well-fed control animals (R4-GAL4>UAS-lacZ). In contrast, R4-GAL4 expression of Flag-Trbl resulted in higher nuclear FoxO-GFP accumulation while Flag-Trbl^SLE/G^ lowered FoxO-GFP to undetectable levels. (D) Analysis of western blot experiments, representing the ratio of density measured for specific bands corresponding to P-Akt divided by density for total Akt, normalized to tubulin. *n*=3 groups of 40 larval fat bodies. One-way ANOVA, Tukey post-hoc. Error bars represent s.d. (E,F) R4-GAL4 expression of Flag-Trbl^SLE/G^ failed to suppress lethality associated with expression of myristoylated HA-tagged Akt (UAS-myr-dAkt, activated Akt) at pupation (E) and at eclosion (F). For each, *n*=3 groups of 40 age-matched larvae. One-way ANOVA, Tukey post-hoc. Error bars represent s.d. Genotypes indicated below each bar. (G-G″) R4-GAL4 co-expression of Flag-Trbl and HA-Slbo results in low levels of nuclear HA-Slbo, which colocalized with Trbl. DAPI overlay is shown in G″. Genotype: R4-GAL4>UAS-Flag-Trbl, UAS-HA-Slbo. (H-H″) R4-GAL4 co-expression of Flag-Trbl^SLE/G^ and HA-Slbo results in increased levels of nuclear HA-Slbo and ectopic Flag-Trbl^SLE/G^ in the nucleus (respective arrows in H and H′). Genotype: R4-GAL4>UAS- Trbl^SLE/G^, UAS-HA-Slbo. (I-I″) R4-GAL4 co-expression of Flag-Trbl and HA-Stg results in low levels of HA-Stg (I′), which colocalizes with Trbl (G) at the cell membrane and in the nucleus. DAPI overlay is shown in I″. Genotype: R4-GAL4>UAS-Flag-Trbl, UAS-HA-String. (J-J″) R4-GAL4 co-expression of Flag-Trbl^SLE/G^ and HA-Stg results in greatly increased levels of HA-Stg throughout the cell. DAPI overlay is shown in J″. Genotype: R4-GAL4>UAS-Flag-Trbl^SLE/G^, UAS-HA-String. n.s., not significant. **P<0.005, ***P<0.0005. Scale bars: 50 µm.

Although Trbl^SLE/G^ bound to and stabilized WT Trbl, it exerted no effect on an HA-tagged Akt that was co-expressed (compare Fig. 3B and C). To examine the effect of Trbl^SLE/G^ on Akt activity, we used western blots to assess Akt phosphorylation in response to Trbl^SLE/G^ expression. We extracted protein from fat body tissue expressing either Trbl RNAi, Flag-Trbl or Flag-Trbl^SLE/G^ and probed with anti-phospho-dAkt S505 sera and anti-pan-Akt sera, respectively (Fig. 5A,D). As shown previously, expression of Trbl RNAi led to increased P-Akt levels while expression of WT Trbl led to reduced P-Akt levels; neither treatment affected total Akt levels detected using a pan-Akt (Fig. 5A,D; Das et al., 2014; Du et al., 2003; Hong et al., 2016; Fischer et al., 2017). In contrast, expression of Flag-Trbl^SLE/G^ resulted in significantly higher P-Akt levels in well-fed larvae (Fig. 5A,D), again without affecting total Akt levels.

To confirm that Trbl^SLE/G^ increases Akt activity, we examined its effects on levels of the Akt target FoxO. On western blots of larval fat body lysates, Trbl expression led to increased levels of FoxO protein compared to a UAS-*lacZ* control (Fig. 5B), consistent with the ability of Trbl to antagonize Akt activity. In contrast, Flag-Trbl^SLE/G^ expression resulted in undetectable levels of FoxO levels (Fig. 5B; Hwangbo et al., 2004). In the fat body, Trbl expression led to increased levels of a FoxO-GFP transgene in nuclei compared to controls, in which FoxO-GFP levels were low and cytoplasmic (Fig. 5C). Expression of Flag-Trbl^SLE/G^ resulted in undetectable levels of FoxO (Fig. 5C). These data are consistent with the ability of Trbl^SLE/G^ to interfere with endogenous Trbl, effectively derepressing Akt activity. Consistent with the opposing effects of WT Trbl and Trbl^SLE/G^ on Akt activity, we observed that while WT Flag-Trbl effectively suppressed myr-dAkt-mediated lethality resulting in escaper adults, Flag-Trbl^SLE/G^ co-expression with myr-dAkt increased lethality at pupation and eclosion stages (Fig. 5E and F, respectively).

The ability of Trbl^SLE/G^ to increase Akt activity without affecting Akt levels led us to test the effect of Trbl^SLE/G^ co-expression on levels of HA-tagged Trbl degrons String (Stg; a Cdc25 phosphatase) and Slbo (a C/EBP transcription factor encoded by the gene *slow border cells*). Co-expression of WT Trbl with either HA-Slbo (Fig. 5H) or HA-Stg (Fig. 5I) led to low levels of HA in the nucleus, consistent with rapid proteasomal turnover of these targets by Trbl. In contrast, when we co-expressed Trbl^SLE/G^ with HA-Slbo (Fig. 5H) or HA-Stg (Fig. 5J), we observed aberrantly high levels of HA staining for both. We note that co-expression of Trbl^SLE/G^ and Slbo-HA resulted in accumulation of Trbl^SLE/G^ in the nuclei of some cells (Fig. 5H, arrow), perhaps due to a stronger affinity of Slbo for Trbl^SLE/G^, a possibility confirmed by yeast two-hybrid assay (Fig. S9). The ability of Trbl^SLE/G^ to increase the stability of three Trbl degrons – Slbo (C/EBP; Fig. 5H), String (Cdc25 phosphatase; Fig. 5J) and Trbl (Fig. 4E) – indicates that the SLE/G mutation dominantly increases the stability of a wide range of Trbl targets during development; in support of this, R4-GAL4 fat body-specific expression of Trbl^SLE/G^ led to decreased larval mass, cell size and delayed pupation compared to WT Trbl (Fig. S5G,H). We conclude that dominant-negative SLE/G forms unproductive heterodimers with endogenous Trbl to block its function, resulting in increased stability and activity of various Trbl substrates.

### Activated Akt recruits WT Trbl to the membrane

**Fig. 6.**
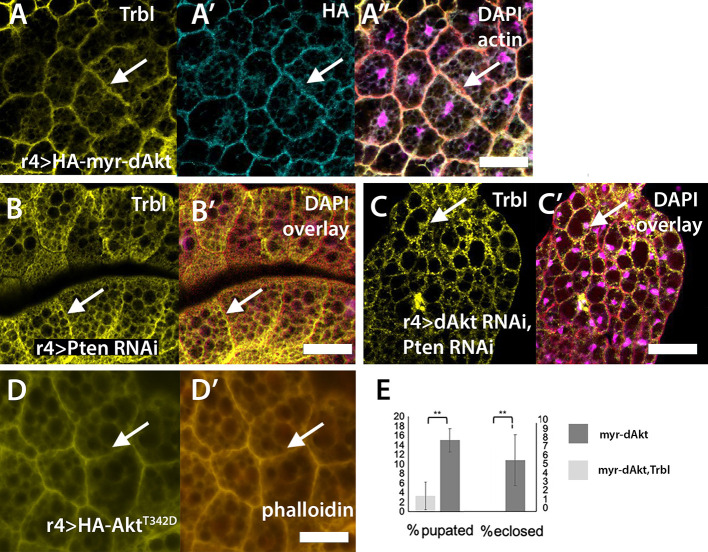
**Trbl protein association with the fat body cell membrane is dependent on conditions that activate Akt.** (A-A″) R4-GAL4 co-expression of HA-Myr-dAkt and UAS-Trbl shows HA-Akt accumulation at the membrane (A′, arrow) sufficient to recruit Trbl protein to the cell membrane (A, arrow), resulting in low Trbl levels in the nucleus. Genotype: R4GAL4>UAS-Trbl, UAS-HA-Akt. A″ is overlay with DAPI staining in magenta. (B,B′) R4-GAL4 co-expression of RNAi to PTEN and Flag-Trbl shows Trbl accumulation at the cell membrane (arrows). DAPI overlay is shown in B′. Genotype: R4GAL4>UAS-Trbl, UAS-PTENi. (C,C′) R4-GAL4 co-expression of Trbl with RNAi to PTEN and RNAi to Akt showed Trbl accumulation in the nucleus (C, arrow). DAPI overlay is shown in C′. Genotype: R4GAL4>UAS-Trbl,UAS-dAkti, UAS-PTENi. (D,D′) R4-GAL4 expression of UAS-HA-dAktT342D, an GAL4-inducible phosphomimetic of Akt, results in endogenous Trbl protein accumulation at the cell membrane (D, arrow). DAPI overlay is shown in D′. Genotype: R4GAL4>UAS-HA-dAktT342D. (E) R4-GAL4 expression of Flag-Trbl suppressed the lethality associated with expression of myristoylated HA-tagged Akt (UAS-myr-dAkt, activated Akt) at pupation and eclosion. G0 is shown as a percentage of larvae pupated. *n*=3 groups of 40 age-matched larvae. One-way ANOVA, Tukey post-hoc. Error bars represent s.d. **P<0.005. Genotype: R4GAL4>UAS-Flag-Trbl, UAS-myr-dAkt. Scale bars: 50 µm.

Trbl^SLE/G^ localization to the membrane was dependent on Akt activity, so we examined the effects of increased Akt signaling on Flag-Trbl localization. To do this, we co-expressed Trbl and an HA-tagged myristoylated Akt (UAS-HA-myr-dAkt), a form of Akt which is localized to the membrane and constitutively activated by Pi3K (Fig. 6A′, arrow; Zheng et al., 2007). HA-myr-dAkt effectively increased Trbl levels at the membrane resulting in a corresponding decrease in nuclear Trbl accumulation (Fig. 6A, arrow; Fig. S6B). Strong lethality associated with fat body expression of UAS-HA-myr-dAkt alone was effectively suppressed by co-expression of UAS-Trbl (expressed as percentage pupated and percentage eclosed, respectively; Fig. 6E), consistent with the notion that Trbl blocks activated Akt activity.

In a second approach, we co-expressed Flag-Trbl and an RNAi transgene to the phosphatase PTEN (UAS-Trbl with UAS-PTEN RNAi and UAS-dAkt RNAi), which leads to increased levels of the phospholipid PIP3 [phosphatidylinositol (3,4,5)-trisphosphate] at the membrane and Akt activation (Perkins et al., 2015). We observed that PTEN^i^ resulted in increased membrane localization of UAS-Flag-Trbl (Fig. 6B, arrow) and a corresponding reduction in nuclear Trbl levels. To determine whether PTEN^i^ localization of Trbl to the membrane requires Akt, we co-expressed Flag-Trbl with UAS-PTEN RNAi and UAS-dAkt RNAi and observed a strong reduction in fat cell size and the accumulation of large lipid droplets (Fig. S6D). Despite the rudimentary appearance of these fat bodies co-expressing Trbl, PTEN^i^ and Akt^i^, we detected Trbl accumulation in the nucleus (Fig. 6C, arrow), suggesting that Trbl protein accumulation at the membrane in response to PTEN RNAi requires Akt. In a third approach to increase Akt activation in well-fed animals, we expressed a Akt phospho-mimetic mutation (an HA-dAkt T342D) and observed increased accumulation of endogenous Trbl at the membrane (Fig. 6D, arrow) with only low nuclear levels. Our data demonstrate that increased Akt is sufficient to localize Trbl to the membrane and supports the notion that Trbl reduces high levels of Akt activity, even in well-fed animals.

### The C-terminal tail of Trbl mediates the influence of the activation loop

Crystallographic analysis of human TRIB1 bound to substrate reveals that the activation loop changes from an ‘out’ to an ‘in’ configuration resulting in release of the C-terminal tail from an intramolecular association with the N-lobe (Jamieson et al., 2018). To understand better the role of the activation loop in promoting Trbl instability and localization in the fat body of well-fed animals, we designed a series of glycine replacements spanning the conserved residues 283-ESLE-286 in the loop (Fig. S8). As shown in Fig. S8B, the glycine replacements E283G (E/GSLE), S284G (ES/GLE) or L285G (ESL/GE) in UAS-Trbl and an SLE/A alanine replacement resulted in levels and localization comparable to Trbl in fat body tissue examined on western blots (Fig. S8B) and tissue (Fig. S8E-G, arrows).

**Fig. 7.**
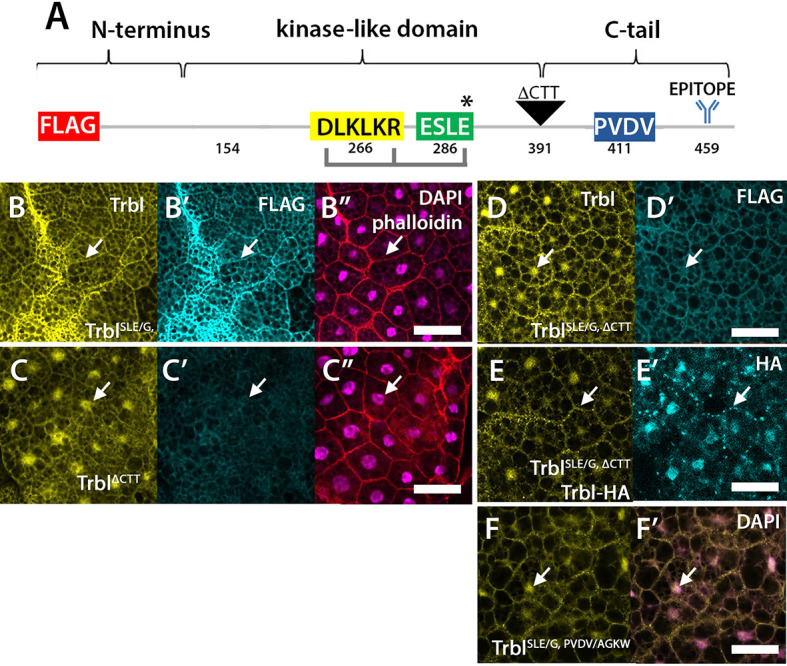
**The C-terminal tail is required for Trbl^SLE/G^ phenotypes.** (A) Map of FLAG-Trbl protein indicating the location of conserved motifs in the kinase-like domain and C-terminal tail. Location of the ELSE/G mutation examined in the experiments shown in this figure is indicated by an asterisk. The location of the C-tail deletion at residue 391 is indicated and the location of the Trbl antisera epitope at residue 459 is indicated. (B-B″) R4-GAL4 expression of Flag-Trbl^SLE/G^ shows that Flag accumulation (B′) parallels the pattern of aberrant Trbl accumulation (B) at the fat body cell membrane (arrows). DAPI and phalloidin overlay is shown in B″. Genotype: R4-GAL4>UAS-Flag-Trbl^SLE/G^. (C-C″) R4-GAL4 expression of Flag-Trbl^ΔCTT^ shows that Flag accumulation (C′) parallels the pattern of normal Trbl accumulation (C) throughout the cell, but mainly in the nucleus (arrows). DAPI and phalloidin overlay is shown in C″. Genotype: R4-GAL4>UAS- Flag-Trbl^ΔCTT^. (D,D′) R4-GAL4 expression of Flag-Trbl^SLE/G, ΔCTT^ shows that Flag (D′) accumulates throughout the cell (mainly in the nucleus as indicated by arrows) with aberrant punctate staining at the cell membrane (arrows). Genotype: R4-GAL4>UAS-Flag-Trbl^SLE/G, ΔCTT^. (E,E′) R4-GAL4 co-expression of Flag-Trbl^SLE/G, ΔCTT^ and Trbl-HA shows HA accumulation throughout the cell with aberrant punctate staining at the cell membrane (arrows). Genotype: R4-GAL4>UAS-Flag-Trbl^SLE/G, ΔCTT^, Trbl-HA. (F,F′) R4-GAL4 expression of Flag-Trbl^SLE/G, PVDV/AGKW^ shows Trbl accumulation (F) throughout the cell with nuclear accumulation (arrows) and aberrant punctate staining at the cell membrane. DAPI overlay in F'. Genotype: R4-GAL4>UAS-Flag-Trbl^SLE/G, PVDV/AGKW^. Scale bars: 50 µm.

These outcomes indicated that the activation loop mutation Trbl^SLE/G^ is unique in its effects on Trbl protein turnover and localization. To determine whether Trbl^SLE/G^ phenotypes require the C-terminal tail, we deleted the tail in both WT Trbl and Trbl^SLE/G^ by inserting a stop codon at residue 391 to generate Flag-Trbl^ΔCTT^ and Flag-Trbl^SLE/G+ΔCTT^, respectively (Fig. 7A) and compared the stability and localization of these transgenes. Tail deletions remove the Trbl antiserum epitope, so we used an anti-Flag antibody, which detects the N-terminal tag on these proteins. On western blots, we observed that the Flag-Trbl^SLE/G+ΔCTT^ double mutant showed reduced levels compared to Flag-Trbl^SLE/G^ (Fig. S8C). In the transgenic fat body, the double-mutant Flag-Trbl^SLE/G+ΔCTT^ accumulated mainly in the nucleus with only weak, punctate accumulation of Flag staining at the membrane (Fig. 7D,D′). We conclude that the C-tail is required for the increased stability conferred by the SLE/G mutation.

To test the ability of the double-mutant Flag-Trbl^SLE/G+ΔCTT^ to recruit Trbl-HA to the fat body cell membrane, we co-expressed both and observed nuclear HA accumulation with aberrant punctate HA staining at the membrane (Fig. 7E). To probe further the requirement of the C-terminal tail for Trbl^SLE/G^ phenotypes, we focused on a PVDV sequence in this domain, which is strongly conserved among drosophilids (Dobens et al., 2021; Fig. 7A). We generated a double-mutant transgene Flag-Trbl^SLE/G+PVDV/AGKW^, which retains the Trbl antiserum epitope and observed mainly nuclear accumulation with only weak, punctate Trbl at the membrane (Fig. 7F). Together with the controls presented in Fig. 7 and Fig. S7, these outcomes indicate that the C-tail, and specifically a conserved PVDV motif, is required for the dominant effects of the Trbl^SLE/G^ mutation, including aberrant membrane localization, reduced instability and the ability to recruit Trbl protein to the membrane.

## DISCUSSION

We have previously shown that, like the mammalian isoform Trib3, fly Trbl binds and inhibits the phosphorylation of Akt to reduce Insulin-like signaling (Das et al., 2014). In this work, we show that Trbl levels increase in the fat body storage organ in larvae subjected to fasting, similar to the effect of fasting on Trbl expression reported in the adult fat body, a distinct organ (Hong et al., 2016). We show here that, in response to fasting, Trbl protein is redistributed from the nucleus to the membrane to facilitate this inhibition of Akt, suggesting that regulation of Trbl translocation mediates its ability to act as a rheostat or dimmer switch to modulate insulin responses in the face of dietary stress.

Our data show also that, even in well-fed animals, Trbl can translocate to the fat body cell membrane in response to increased Akt activity, presumably to maintain proper levels of signaling. Consistent with this, expression of an activation loop mutation, Trbl^SLE/G^, that dominantly blocks Trbl function results in increased Akt activity, as measured by increased phosphorylation of Akt and reduced FoxO levels, leading to a striking localization of Trbl proteins to the membrane. Other explanations for the effects of Trbl^SLE/G^ on Akt derepression are formally possible: for example, because mammalian Trib isoforms can both activate and inhibit Akt depending on the isoform and the context (Naiki et al., 2007; Keeshan et al., 2010; Foulkes et al., 2018; Shen et al., 2021; Singh et al., 2023), it is possible that the SLE/G mutation uncovers a dual role for the single *Drosophila* isoform to either activate or block Akt phosphorylation depending on the context. However, the notion that expression of Trbl^SLE/G^ forms inactive Trbl^SLE/G^-Trbl heteromultimers to block Trbl function retains an attractive simplicity to explain not only the increased Akt activity observed, but also the increased stability of Trbl degrons Slbo (C/EBP), String (cdc25 phosphatase) and Trbl itself. Even the strong membrane localization of Trbl^SLE/G^ can be attributed to an increase in Akt activity because (1) it can be reversed by expression of both Akt RNAi and other transgenes that reduce Akt activity and (2) increased Akt activity is sufficient for membrane localization of WT Trbl. A recent paper describing the effect of mouse COP1 E3 ligase mutations on Trib2 levels (Sunami et al., 2024) indicates that rapid turnover of Trib adaptor proteins is conserved (Qiao et al., 2013; Sakai et al., 2016; reviewed by Salomè et al., 2015), and recent identification of nanobodies that bind human TRIB2 and stabilize a TRIB2-TRIB2 dimeric conformation may explain the ability of the Trbl^SLE/G^ mutation to bind, stabilize and interfere with the ability of endogenous Trbl to interact with targets (Jamieson et al., 2022). Thus, in the fat body Trbl^SLE/G^ expression binds, stabilizes and inactivates endogenous Trbl and its localization to the membrane is a phenotype downstream of the resulting increased Akt activity.

The structure of human TRIB1 in the absence of substrate reveals an activation loop in the extended ‘out’ configuration, which stabilizes the pseudokinase spine to maintain an intramolecular N-lobe binding site for the CTT. Coincident with substrate binding (Murphy et al., 2015; Jamieson et al., 2018), the activation loop swings to an ‘in’ position to both stabilize substrate association and release the CTT, which binds an E3 ligase leading to proteasomal degradation of the substrate. For Trbl, replacement of E286 with the small amino acid glycine likely changes the flexibility of the loop dramatically (Yan and Sun, 1997) to alter the functions of the protein. Although the striking Trbl^SLE/G^ phenotypes are not seen in other site-specific mutations scanning the activation loop (Fig. S8, Table S1), the strong conservation of the entire ESLE motif predicts important functional roles in other tissues or contexts. Recent demonstration that TRIB2 stability is sensitive to small molecule kinase inhibitors with consequent effects on Akt signaling (Foulkes et al., 2018) points to the functional importance of activation loop dynamics that may serve as a target for pharmacological intervention (Smith et al., 2021). The dependence of Trbl stability on the C-terminal tail recalls the role of specific sequences in the N-terminal region of the TRIB2 protein that confer tissue-specific turnover mediated by the combination of p70 S6 kinase phosphorylation and binding of the E3 ligase SMURF1 (Wang et al., 2013b). Thus, the SLE/G mutation may interfere with interactions of a yet to be identified kinase and/or E3 ligase that targets Trbl for turnover.

Trbl shuttling from the nucleus to the fat body cell membrane to inhibit Akt activity to modulate insulin responses in response to larval fasting resembles the reverse translocation away from the membrane that the *Drosophila* insulin receptor complex undergoes in response to reduced mechanical cell stress associated with larval inactivity (Kim et al., 2018). These diametrically opposed translocations of insulin signaling agonist, on the one hand, and antagonist on the other suggest a coordinated arthropod strategy to dial down insulin responses to varied environmental cell stresses. The regulated nuclear entry of mouse Trib1 via binding of the CTT to COP1 E3 ubiquitin ligase suggests more broadly that the subcellular distribution of this family of adaptors is tightly regulated among cellular compartments, and the control this exerts on their abilities to interact with various substrates will add to the plasticity of their functions (Kung and Jura, 2019).

## MATERIALS AND METHODS

### *Drosophila* strains

Stocks used in this study include: (1) *Canton S*, (2) UAS-*lacZ* (*P*[+mC]=UAS-lacZ.NZ}*20b*; Indiana Stock Center, ISC #3955), (3) UAS-HA-myr-Akt [*P*(UAS-myr-Akt1.DeltaPH)*3*; ISC #80935], (4) UAS-InRK1409A (*P*[+mC]=UAS-InR.K1409A; ISC #8253), (5) UAS-InR RNAi [*y1 v1; P*(TRiP.JF01482)*attP2*; ISC #31037], (6) UAS-InR [*P*(UAS-InR.Exel)*2*; ISC #8267], (7) UAS-PI3K92EA2860C (*P*[+mC]=UAS-Pi3K92E.A2860C}*1*; ISC #8288), (8) UAS-PI3K92E [*y1 w1118; P*(UAS-Pi3K92E.Exel)*2*; ISC #8286], (9) UAS-PTEN RNAi (*y*[1] *v*[1]*; P*[+t7.7] v[+t1.8]=TRiP.HMS00044}*attP2*; ISC #33643) [+t7.7], (10) UAS-PI3K RNAi [*y1 v1; P*(TRiP.JF02770)*attP2/TM3, Sb1*; ISC #27690], (11) UAS-Akt RNAi (*y*[1] *v*[1]*; P*[+t7.7]v[+t1.8]=TRiP.HMS00007}*attP2*; ISC #33615), (12) tGPH (*Sco/CyO; P*[+mC]=tGPH}*4/TM3*; ISC #8164), (13) w; PBac(y[+mDint2] w[+mC]=dfoxo-GFP.FLAG)VK00037 (ISC #38644), (14) UAS-Akt RNAi (y[1] v[1]; P(y[+t7.7] v[+t1.8]=TRiP.HMS06047)attP40/CyO; ISC #82957), (15) P(ppl-GAL4.P)2 (ISC #58768), (16) P(UAS-chico.C)3 (ISC #93138), (17) P(UAS-S6k.STDE)3 (ISC #6913), (18) y[1] w[*]; P(w[+mC]=UAS-Tsc1.T)3, P(w[+mC]=UAS-gig.T)3 (ISC #80576), (19) y[1] w[*]; P(AyGAL4)25 P(w[+mC]=UAS-lacZ.B)Bg4-1-2/CyO, y[+] (ISC #4409), (20) UAS-Trbl RNAi [*w1118; P*(GD11640)*v22114*; Vienna *Drosophila* Research Center; Dietzl et al., 2007].

The following stocks were obtained from the Zurich ORFeome Project (Bischof et al., 2013): (1) UAS-Trbl-3XHA [*M*(UAS-Trbl.ORF.3XHA)*ZH-86Fb*], (2) UAS-Akt-3XHA [*M*(UAS-Akt1.ORF.3XHA)*ZH-86Fb*], (3) UAS-Slbo-3XHA [*M*(UAS-slbo.ORF.3xHA.GW)*ZH-86Fb*] and (4) UAS-Stg-3XHA [*M*(UAS-stg.ORF.3xHA)*ZH-86Fb*]. R4-GAL4 (y^1^ w*; P(r4GAL4)*3*] was a generous gift from Dr Laura Musselman (Binghampton University, NY, USA) and UAS-dAktT342D was a generous gift from Dr Michelle Bland (University of Virginia, VA, USA) (Yu et al., 2019).

### Construction of transgenes

The design and generation of UAS-FLAG-Trbl, UAS-FLAG-Trbl^FLCR/A^, UAS-FLAG-Trbl^D/NLK^ and UAS-FLAG-Trbl^SLE/G^ stocks were described previously (Masoner et al., 2013).

Trbl-GFP knock-in deleting the 5′-UTR and the first exon was built using an upstream Crispr guide (gcttcttccgaccgcgtggatgg; NT_037436 REGION:20401634/20401612 from the genome assembly) located right outside of Tribbles gene and the downstream Crispr guide (acgtacgccttgcgttcatt; NT_037436 REGION:20400060/20400079) located in exon 1. The resulting deletion of 1555 bp included the full 5′-UTR and the first exon of the *trbl* gene from NT_037436 REGION:20401634 to NT_037436 REGION:20400079 and was replaced by a GFP selection cassette which carried multiple stop codons. This insertion of GFP was designed to generate a null *trbl* allele.

The following stocks were generated for this work using the Q5 Site Directed Mutagenesis Kit (NEB) using pUASTattB-Flag-Trbl as template (Masoner et al., 2013). Mutated codons are shown in bold: UAS-FLAG-Trbl^L285G^ forward primer 5′-GTATGAATCA**GCC**GAAGGCTCAATGATCCTCGAC-3′, reverse primer 5′-TGCAGTTTCGTTCTGGCC-3′; UAS-FLAG-Trbl^R269A^ forward primer 5′-ACTGCAGTATGAATCACTG**GCC**-3′, reverse primer 5′-GGGACAGAGTCCTTCCCTCGT-3′; UAS-FLAG-Trbl^E283G^ forward primer 5′-ACTGCAGTAT**GCC**TCACTGGAAGGCTCAATG-3′, reverse primer 5′-TTCGTTCTGGCCTCGTCG-3′; UAS-FLAG-Trbl^S284G^ forward primer 5′-GCAGTATGAA**GCC**CTGGAAGGCTC-3′, reverse primer 5′-AGTTTCGTTCTGGCCTCG-3′. These same primers were used to introduce the SLE/G mutation into pUAS-Trbl-HA (a kind gift of Johannes Bischof, FlyORF stock center).

UAS-myr-Trbl was generated using the forward primer 5′-AACCCGGAAGACGACGCGGCTATGGATTACAAGGATGAC-3′ and reverse primer 5′-CGAGGAGCACCAGCAGCCCATGAATTCCCAATTCCCTATTC-3′. To generate UAS-TrblΔC and UAS-TrblSLE/G+ΔC we used on the respective templates forward primer 5′-TGAATCACTGGGAGGCTCAATGA-3′ with reverse primer 5′-TACTGCAGTTTCGTTCTG-3′ (Z.F. and L.L.D., unpublished) to introduce a stop codon at residue 39. Primer pairs used to introduce other Trbl mutations are listed in Table S1.

### Tissue preparation

For experiments comparing Trbl protein levels and distribution in age-matched larvae, males and females were mated for 3 days in egg collection chambers on standard MYCM fly food at 25°C and food was replaced daily. On the fourth day, eggs were collected from these crosses on fresh food plates by changing plates every hour to guarantee developmental synchronicity. Crosses designed to express UAS-transgenes in the larval fat body using the GAL4-UAS system were performed at 29°C to increase GAL4 production. Crosses and collection of Canton S larvae and Trbl-CRISPR-GFP larvae were performed at 25°C. The following day, 60 emerged first instar larvae were transferred from each plate to fresh plates at 24 h after egg deposition (AED). Using this approach, each food plate contained the same number of larvae and the larvae within an individual plate were developmentally synchronous.

For fasting, third instar larvae were collected at 96, 101 and 111 h AED, and for the fasted group larvae were transferred to an empty vial containing tissue wetted with distilled water for either 5 h (96 h fed+5 h fasted) or 15 h (96 h fed+15 h fasted). Twenty larvae were collected at these fed/fasted time points. After this, larval fat bodies were collected and processed according to standard larval fat body dissection and immunostaining, described below. For fat body cell Flp-out clone production, larvae of the genotype hs-FLP/+; P(AyGAL4)25 P(w[+mC]=UAS-lacZ.B)Bg4-1-2/+; P[+t7.7]v[+t1.8]=TRiP.HMS00007}attP2 (UAS-Akt RNAi) were reared at 30°C and clones detected by *lacZ* expression according to the approach described by Kim et al. (2018).

### Immunofluorescence

Twenty mid-third instar wandering larvae were washed in fresh PBS, then transferred to fresh ice-cold PBS for dissection. Larval fat bodies were partially dissected and all other internal organs were removed, leaving only the cuticle and attached fat body. These were fixed in 4% paraformaldehyde (Thermo Fisher Scientific) in PBS for 15 min and washed three times in PBS with 0.1% Triton X-100 (PBST; Thermo Fisher Scientific). Tissue was blocked for 1 h in PBST+5% bovine serum albumin (Thermo Fisher Scientific) and incubated overnight at 4°C with primary antibody: chicken anti-Trbl (1:1000; Masoner et al., 2013), rabbit anti-dFoxO (1:5000; Hwangbo et al., 2004), mouse anti-HA (1:1000; 3724S, Cell Signaling Technology), rabbit anti-FLAG (1:1000; 14793S, Cell Signaling Technology), rabbit anti-pan-Akt (1:1000; 4691S, Cell Signaling Technology), mouse-anti β-galactosidase (1:200; JIE7, Developmental Studies Hybridoma Bank) and mouse anti-GFP (1:500; 8-1E7, Developmental Studies Hybridoma Bank). The following day, fat bodies were washed twice in PBST and incubated for 1 h at room temperature with TRITC-conjugated phalloidin (1:400; P1951, Sigma-Aldrich) in PBST. Following this, fat bodies were washed once in PBST and incubated for 2 h at room temperature in appropriate fluorescent secondary antibody at 1:200 in PBST+normal goat serum (Thermo Fisher Scientific). Alexa Fluor secondary antibodies were obtained from Thermo Fisher Scientific and used at 1:200: goat anti-chicken 488 (Invitrogen, A11009), goat anti-rabbit 546 (Invitrogen, A11012), goat anti-chicken 647 (Invitrogen, A21449), goat anti-mouse 546 (Invitrogen, A11005), goat anti-mouse 647 (Invitrogen, A21244). Next, fat bodies were washed twice in PBST and incubated for 30 min in DAPI (1:1000; Sigma-Aldrich) in PBS. Finally, fat bodies were washed once in PBST and mounted on glass slides in 50% glycerol/PBS and visualized on an Olympus Fluoview 300 confocal laser-scanning microscope (CLSM). Images were prepared with ImageJ.

For analysis of Trbl protein subcellular distribution, Trbl, phalloidin and DAPI distribution and intensity in 90 larval fat body cells were analyzed using the ImageJ Plot Profile plugin. Plot Profile intensities were averaged and graphed using Microsoft Excel.

### Pupation/eclosion analysis

Analysis of pupation and eclosion rates were performed by setting crosses at 29°C and collecting 40 age-matched larvae at 72 h AED. These larvae were transferred to fresh food vials and 3 days later the number of pupal cases was documented. This experiment was repeated three times, and statistical analysis was performed using one-way ANOVA and Tukey post-hoc (GraphPad Prism). The experiments analyzing eclosion were performed exactly as above, except vials were analyzed 5 days after transfer and eclosed flies were counted.

### Western blot

Western blot experiments were performed by collecting 20 larval fat bodies, homogenizing on ice in RIPA buffer with protease inhibitor cocktail (Roche), and spun down for 30 min at 4°C. Supernatant was collected, an aliquot was removed for BCA analysis (Pierce) to ensure equivalent loading, and 5× SDS buffer was added to the supernatant. Samples were boiled for 5 min at 95°C and DTT (Sigma-Aldrich) was added to 100 mM. Samples were loaded onto 10% polyacrylamide gels and electrophoresed for 1.5 h at 100 V. Next, semi-dry transfer was performed using PVDF membrane (Millipore) and according to manufacturer protocols (Abcam). Semi-dry transfer was performed at 126 mA for 45 min. Next, PVDF membranes were blocked for 1 h in TBST (Tris-buffered saline with Tween 20) with 3% bovine serum albumin and incubated overnight at 4°C in primary antibody. Primary antibodies used were: chicken anti-Tribbles (1:2000; Masoner et al., 2013), rabbit anti-FLAG (1:1000; 14793, Cell Signaling Technology), mouse anti-alpha tubulin (1:2000; Clone ID 4A1, Developmental Studies Hybridoma Bank), rabbit anti-phospho-dAkt S505 (1:1000; 4060, Cell Signaling Technology) and rabbit anti-pan-Akt (1:1000; 9272, Cell Signaling Technology. Next, PVDF membranes were washed three times for 5 min in TBST and incubated for 2 h at room temperature in appropriate horseradish peroxidase (HRP)-conjugated secondary antibody. Secondary antibodies used were: goat anti-rabbit HRP (1:2000; 7074, Cell Signaling Technology), goat anti-mouse HRP (1:2000; 91196, Cell Signaling Technology) and goat anti-chicken HRP (1:5000; ab112821, Abcam). Following this, PVDF membranes were washed three times for 5 min each wash in TBST and incubated in ECL plus chemiluminescent substrate (Pierce) for 5 min. Chemiluminescent signals were detected using ChemiDoc (Bio-Rad) and analyzed using one-way ANOVA followed by Tukey post-hoc (GraphPad Prism).

### Yeast two-hybrid interaction analysis

Construction of pDEST32-Trbl and pDEST32-Trbl^SLE/G^ was described previously (Masoner et al., 2013). pDEST22-dAkt was constructed by PCR amplification of an Akt cDNA (Origene) to add flanking attB1 and attB2 sites. Using the Gateway Technology system (Invitrogen), this Akt fragment was cloned into pDONR-221 and then into the destination vector pDEST22 and confirmed by DNA sequencing. Yeast media preparation, plasmid DNA transformation into MaV203 yeast, and plating were described previously (Masoner et al., 2013).

## Supplementary Material



10.1242/develop.204493_sup1 Supplementary information
