# Supplementary material for: The Drosophila pseudokinase Tribbles translocates to the fat body membrane in response to fasting to modulate insulin sensitivity
Source: Development. 2025 Apr 28;152(8):dev204493. doi: 10.1242/dev.204493 (PMC12070071; doi:10.1242/dev.204493)
Supplement: Supplementary information [file develop-152-204493-s1.pdf]

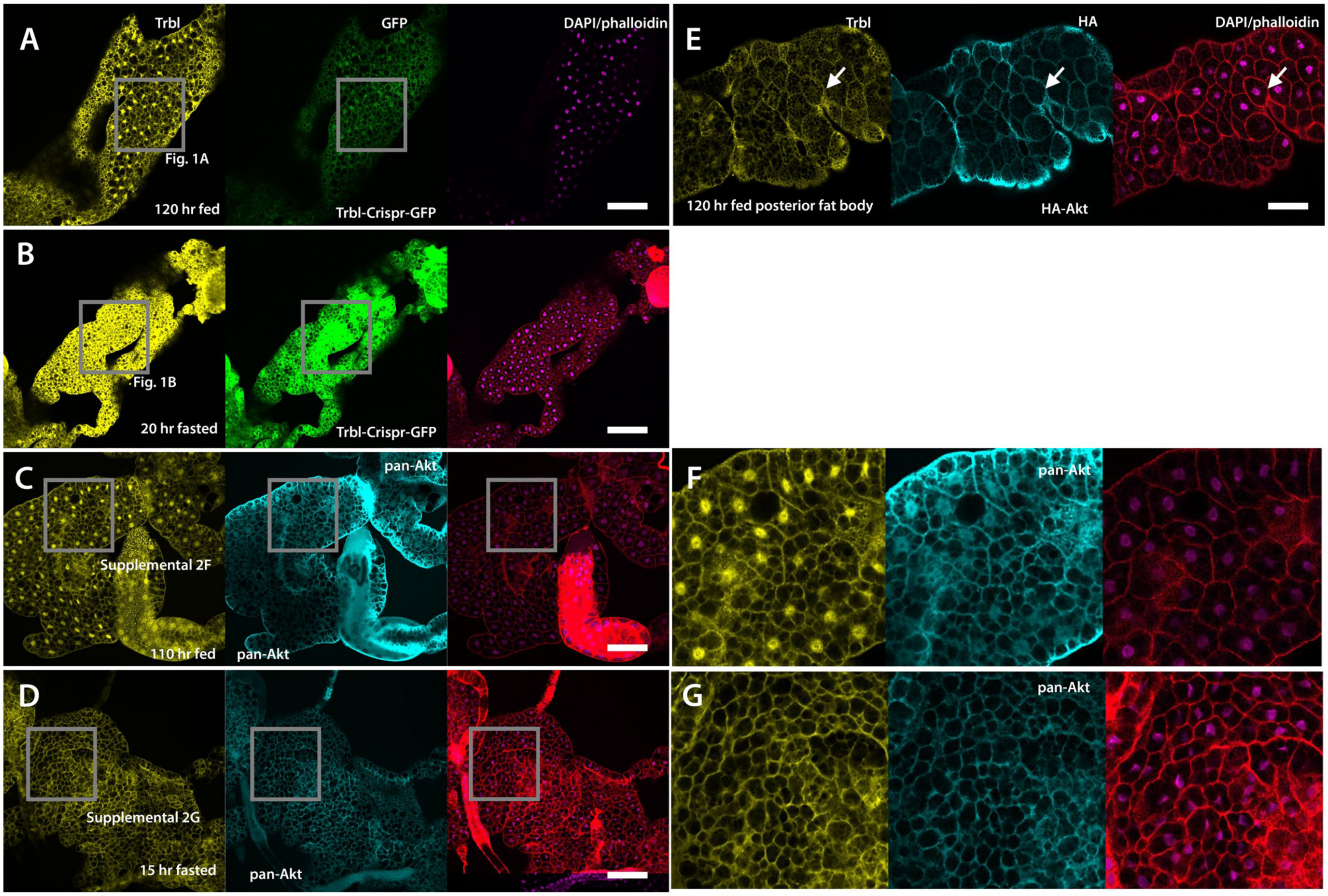

Fig. S1. Tribbles protein and gene expression effects in fat body organs

A-A”. Whole organ fat body expressions of Trbl protein (yellow, A) and a Trbl-GFP reporter gene (green, A’) are low at 120 hr AED (after egg deposition) in fed larvae (DAPI and phalloidin staining in purple and red, respectively, A”). Genotype: Trbl-GFP/TM3Sb. Region in box corresponds to Fig. 1A. Scale bar in this and all panels for Supplemental figures is 100um.

B-B”. Expressions of Trbl protein (yellow, B) and a Trbl-GFP reporter gene (green, B’) increases at 111 hr in larvae that were fasted for 15 hr (DAPI and phalloidin staining in purple and red, respectively, B”). Genotype: Trbl-GFP/TM3Sb. Region in box corresponds to Fig. 1B.

C,C”. In well-fed animals, R4-GAL4 expression of UAS-Trbl in well-fed animals showed that distribution of Trbl protein (yellow, C) is stronger in the nucleus (arrow) and accumulates at lower levels in the cytoplasm and cell membrane at 110 hr AED. The distribution of dAkt detected using a pan-Akt antibody revealed a wide distribution of staining in the nucleus, cytoplasm and cell membrane (blue, C’). DAPI and phalloidin staining in purple and red, respectively, C”). Genotype: R4-GAL4/UAS-Trbl. Region in box corresponds to Fig. 2A.

D,D”. In fasted animals (following 15 hr of fasting), R4-GAL4 expression of UAS-Trbl (yellow, D) in the fat body resulted in low Trbl levels in the nucleus (D) and higher levels at the cell membrane, with pan-dAkt staining also stronger at the cell membrane (D’). DAPI and phalloidin staining in purple and red, respectively (D”). Genotype: R4-GAL4/UAS-FlagTrbl. Region in box corresponds to Fig. 2B.

E,E”. R4-GAL4 expression of UAS-Flag-Trbl in well-fed animals showed Trbl protein distribution (yellow, A) is stronger in the nucleus (arrow) and accumulates at lower levels in the cytoplasm and cell membrane at 110 hr AED. The distribution of dAkt detected using a pan-Akt antibody revealed staining in the nucleus, cytoplasm and cell membrane (blue, A’). DAPI and phalloidin staining in purple and red, respectively, A”). Genotype: R4-GAL4>UAS-Trbl. Scale bar in this and all panels is 50um.

F,F”. R4-GAL4 expression of UAS-Flag-Trbl in well-fed animals showed Trbl protein distribution (yellow, A) is stronger in the nucleus (arrow) and accumulates at lower levels in the cytoplasm and cell membrane at 110 hr AED. The distribution of dAkt detected using a pan-Akt antibody revealed staining in the nucleus, cytoplasm and cell membrane (blue, A’). DAPI and phalloidin staining in purple and red, respectively, A”). Genotype: R4-GAL4>UAS-Trbl. Scale bar in this and all panels is 50um.

G,G”. R4-GAL4 expression of UAS-Trbl in fasted animals (following 15 hr of fasting) resulted in low Trbl levels in the nucleus (yellow, A) and higher levels at the cell membrane (arrow), with pan-dAkt staining also stronger at the cell membrane (B’). DAPI and phalloidin staining in purple and red, respectively (B”). Genotype: R4-GAL4>UAS-Trbl.

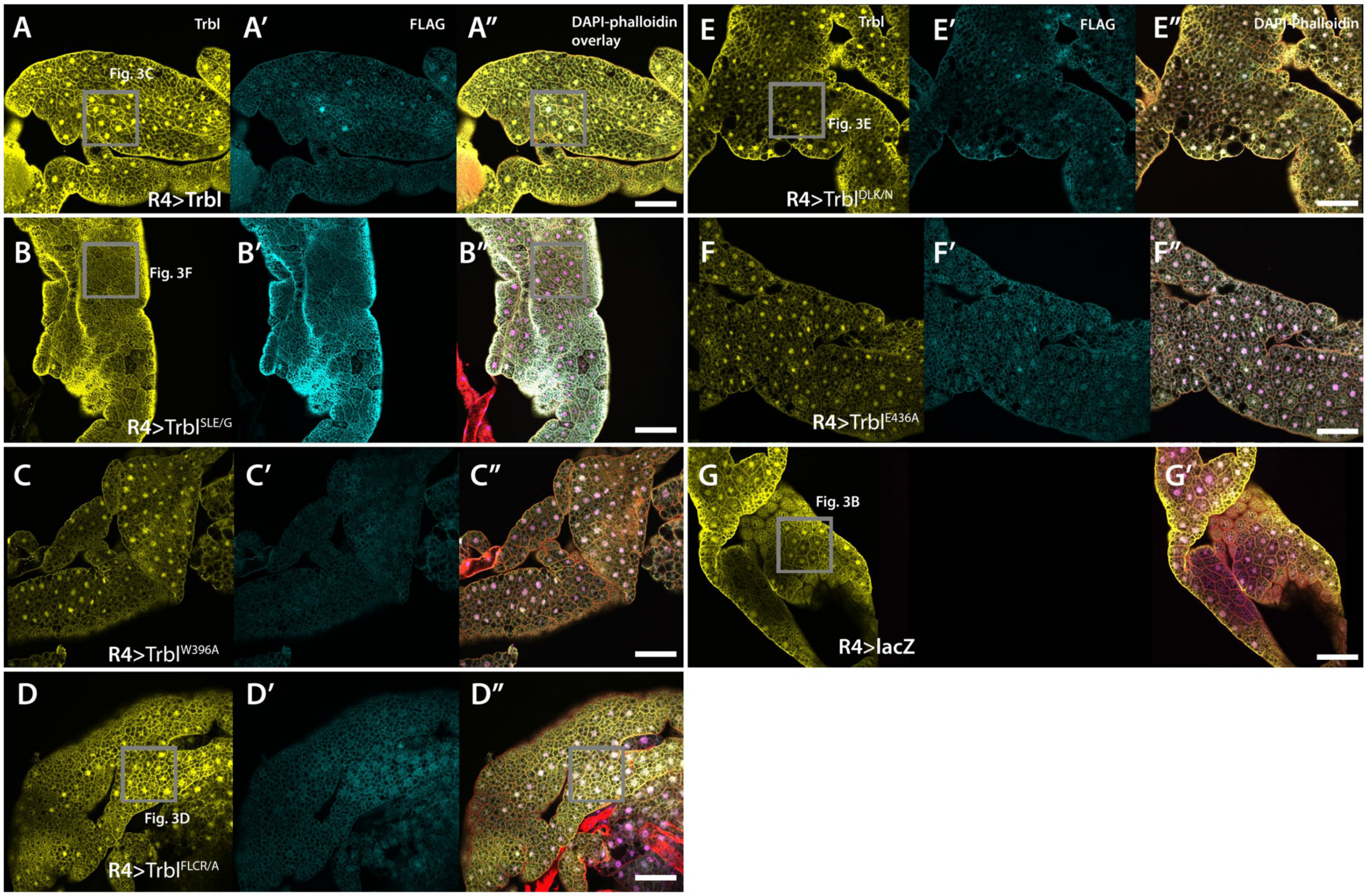

Fig. S2. The activation loop mutation Trbl$^{SLE/G}$ results in Trbl membrane accumulation

A-A”. R4-GAL4 expression of Flag-Trbl detected by antisera to Trbl (A) and Flag (A’) detected similar distribution. DAPI and phalloidin staining in purple and red, respectively (A”). Region in box corresponds to Fig. 3C. Genotype: R4-GAL4>Flag-Trbl. Scale bar in this and all panels for Supplemental figures is 100um.

B-B”. R4-GAL4 expression of Flag-Trbl SLE/G resulted in low levels of Trbl and Flag staining (B and B’, respectively) in the nucleus with strongly increased accumulation at the cell membrane (arrow). DAPI and phalloidin staining in purple and red, respectively (B”). Region in box corresponds to Fig. 3F. Genotype: R4-GAL4>Flag-Trbl SLE/G

Development • Supplementary information

C-C”. R4-GAL4 expression of Flag-TrblW368A detected by antisera to Trbl (C) and Flag (C’) reveals similar distribution. DAPI and phalloidin staining in purple and red, respectively (C”). Genotype: R4-Flag-TrblW368A

D-D”. R4-GAL4 expression of Flag-TrblFLCR/A detected by antisera to Trbl (D) and Flag (D’) reveals similar distribution. DAPI and phalloidin staining in purple and red, respectively (D”). Region in box corresponds to Fig. 3D. Genotype: R4-GAL4>Flag-TrblFLCR/A

E-E”. R4-GAL4 expression of Flag-TrblDLK/N detected by antisera to Trbl (E) and Flag (E’) reveals similar distribution. DAPI and phalloidin staining in purple and red, respectively (D”). Region in box corresponds to Fig. 3E. Genotype: R4-GAL4>Flag-TrblDLK/N

F-F”. R4-GAL4 expression of Flag-TrblE436A detected by antisera to Trbl (F) and Flag (F’) reveals similar distribution. DAPI and phalloidin staining in purple and red, respectively (F”). Genotype: R4-GAL4 expression of Flag-TrblE436A

G,G’. Endogenous Trbl accumulates in nucleus (arrow), with lower levels in cytoplasm and at the membrane. Region in box corresponds to Fig. 3B. Genotype: Canton S

I-I”. Ppl-GAL4 driving expression of Flag-Trbl$^{SLE/G}$ detects Trbl accumulation strongly at the cell membrane. DAPI/phallodin overlay in H’. Genotype: Ppl-GAL4>Flag-Trbl SLE/G

Development • Supplementary information

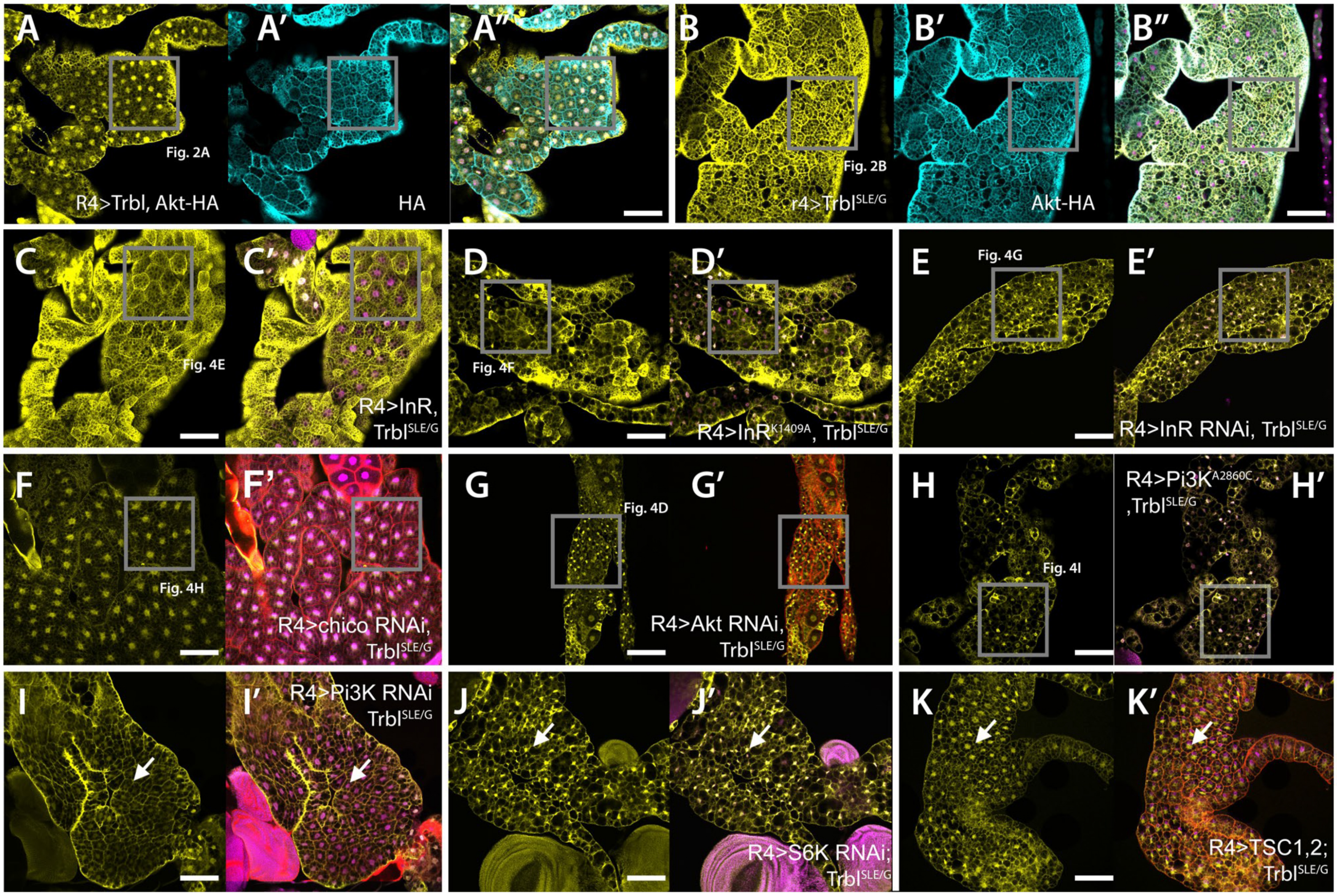

Fig. S3. Membrane association of Trbl$^{SLE/G}$ depends on level of dAkt activation

A-A”. R4-GAL4 co-expression of Trbl and HA-tagged dAkt revealed Trbl protein levels are high in the nucleus and accumulated at lower levels in the cytoplasm and cell membrane while HA-dAkt is strong at the cell membrane. DAPI overlay in A”. Region in box corresponds to Fig. 2A. Genotype: R4-GAL4>UAS-Trbl, UAS-HA-dAkt. Scale bar in this and all panels for Supplemental figures is 100um.

B-B”. R4-GAL4 co-expression of Trbl$^{SLE/G}$ and HA-tagged dAkt showed Trbl protein accumulates at the cell membrane (B) while HA-dAkt remains unchanged in localization and levels at the cell membrane (B’). DAPI overlay in B”. Region in box corresponds to Fig. 2B. Genotype: R4GAL4>UAS-Flag-Trbl$^{SLE/G}$, UAS-HA-dAkt

Development • Supplementary information

C,C’. R4-GAL4 co-expression of UAS-InR (WT insulin receptor) and Flag-Trbl$^{SLE/G}$ resulted in strong accumulation of Trbl at the membrane. DAPI overlay in C’. Region in box corresponds to Fig. 2E. Genotype: R4-GAL4>UAS-InR, UAS-Flag-Trbl$^{SLE/G}$

D,D’. R4-GAL4 co-expression of a UAS-regulated dominant negative version of the insulin receptor (InRK1409A) and Flag-Trbl$^{SLE/G}$ resulted in reduced accumulation of Trbl at the membrane (compared to Flag-Trbl$^{SLE/G}$ alone) and the appearance of nuclear Trbl. DAPI overlay in D’. Region in box corresponds to Fig. 2F. Genotype: R4-GAL4>UAS-InRK1409A, UAS-Flag-Trbl$^{SLE/G}$

E,E’. R4-GAL4 co-expression of an RNAi transgene to InR and Flag- Trbl$^{SLE/G}$ resulted in reduced levels of Trbl accumulation at the membrane and variable nuclear accumulation. DAPI overlay in E’. Region in box corresponds to Fig. 2G. Genotype: R4-GAL4>UAS-*InR* RNAI, UAS-Flag-Trbl$^{SLE/G}$

F,F’. R4-GAL4 co-expression of an RNAi transgene to *chico* which encodes the IRS (insulin receptor substrate) and Flag-Trbl$^{SLE/G}$ resulted in reduced levels of Trbl accumulation at the membrane (compared to Flag-Trbl$^{SLE/G}$ alone) and increased nuclear accumulation. DAPI overlay in F’. Genotype: R4-GAL4>*chico*RNAi, Flag- Trbl$^{SLE/G}$

G,G’. Co-expression of a UAS-regulated RNAi transgene to dAkt and Flag-Trbl$^{SLE/G}$ a resulted in reduced accumulation of Trbl at the membrane and the appearance of ectopic nuclear Trbl. Phalloidin overlay in G’. Region in box corresponds to Fig. 2D. R4GAL4>UAS-Flag-Trbl$^{SLE/G}$, UAS-dAkt RNAi

H,H’. R4-GAL4 co-expression of UAS-regulated dominant negative version of PI3K (PI3KA2860C) and Flag-Trbl$^{SLE/G}$ resulted in both reduced accumulation of Trbl at the membrane and the appearance of nuclear Trbl accumulation. DAPI overlay in H’. Genotype: R4-GAL4>UAS-PI3KA2860C, Flag-Trbl$^{SLE/G}$

I,I’. R4-GAL4 co-expression of an RNAi transgene to *Pi3K* and Flag- Trbl$^{SLE/G}$ resulted in reduced levels of Trbl accumulation at the membrane and increased nuclear accumulation. DAPI overlay in I’. R4-GAL4>UAS-PI3K RNAi, Flag- Trbl$^{SLE/G}$

J,J’. R4-GAL4 co-expression of an RNAi transgene to *S6K* and Flag- Trbl$^{SLE/G}$ resulted in reduced levels of Trbl accumulation at the membrane and increased nuclear accumulation. DAPI overlay in I’. Genotype: R4-GAL4>S6KRNAi, Flag- Trbl$^{SLE/G}$

K,K’. R4-GAL4 co-expression of an RNAi transgenes to both TSC1 and TSC2 and Flag- Trbl$^{SLE/G}$ resulted in reduced levels of Trbl accumulation at the membrane and increased nuclear accumulation. DAPI overlay in I’. Genotype: R4-GAL4>UAS-TSC1, UAS-TSC2, Flag- Trbl$^{SLE/G}$

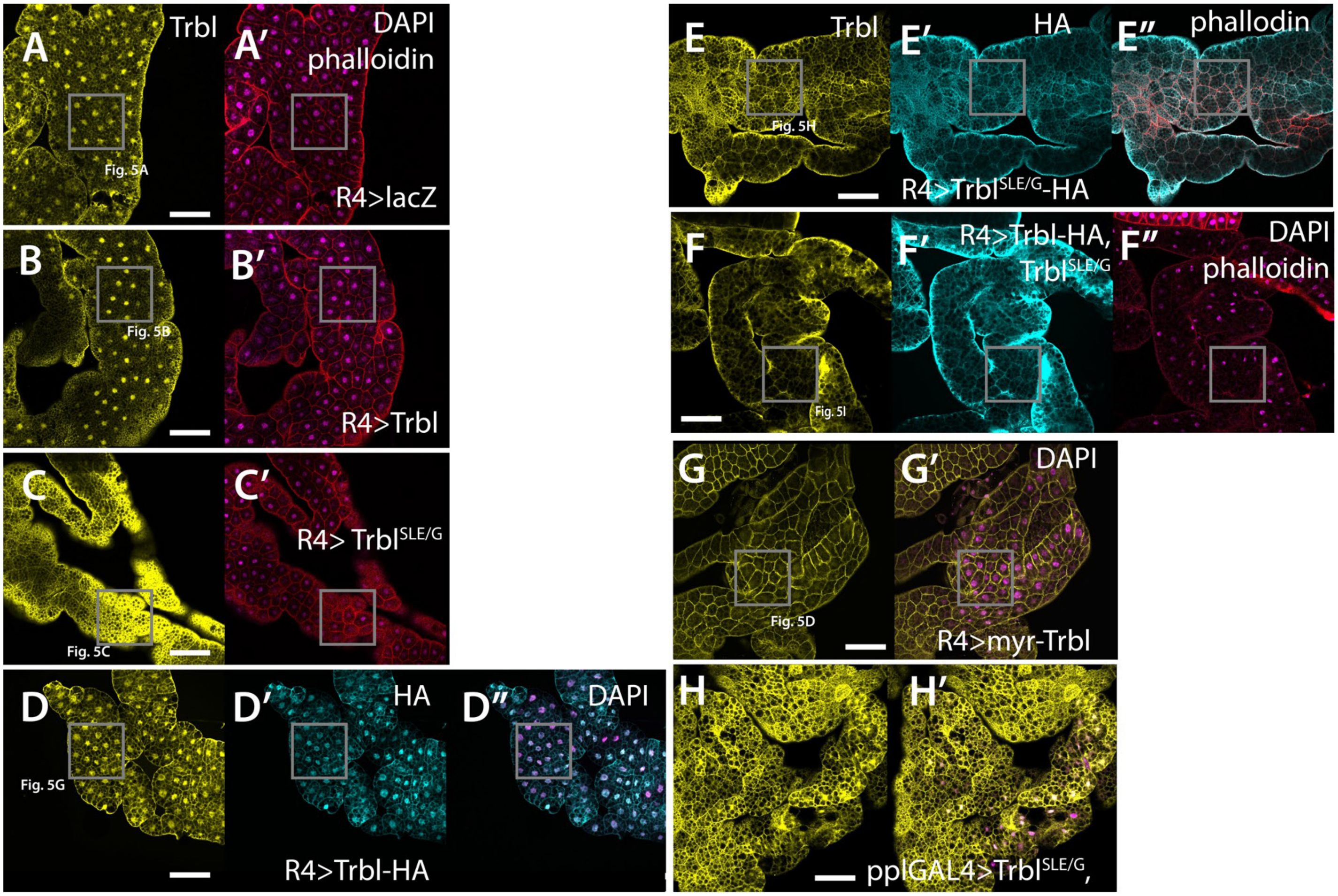

Fig. S4. Trbl$^{SLE/G}$ stabilizes Trbl complexes at the fat body membrane

A,A'. R4-GAL4 driving expression of UAS-lacZ. DAPI and phalloidin staining in A". Region in box corresponds to Fig. 5A. Genotype: R4-GAL4>UAS-lacZ. Scale bar in this and all panels for Supplemental figures is 100um.

B,B'. R4-GAL4 driving expression of UAS-Flag-Trbl. DAPI and phalloidin staining in B". Region in box corresponds to Fig. 5B. Genotype: R4-GAL4>UAS-Flag-Trbl

C,C'. R4-GAL4 driving expression of UAS-Flag-Trbl$^{SLE/G}$. DAPI and phalloidin staining in C". Region in box corresponds to Fig. 5C. Genotype: R4-GAL4>UAS-Flag-Trbl$^{SLE/G}$

D-D". R4-GAL4 expression of C-terminally tagged Trbl-HA detects fat body cell distribution of HA strongly in the nucleus and more weakly in the cell membrane and cytoplasm. DAPI and phalloidin staining in D". Region in box corresponds to Fig. 5G. Genotype: R4-GAL4>UAS-Trbl-HA

E-E". R4-GAL4 expression of C-terminally tagged Trbl$^{SLE/G}$-HA detects fat body cell distribution of HA strongly at the cell membrane (arrow), and variably in the nucleus. DAPI and phalloidin staining in E". Region in box corresponds to Fig. 5H. Genotype: R4-GAL4>Trbl$^{SLE/G}$-HA

Development • Supplementary information

F-F”. R4-GAL4 driving co-expression of Flag-Trbl$^{SLE/G}$ and Trbl-HA detects HA strongly at the cell membrane. DAPI/phallodin in F”. Genotype: R4-GAL4>Flag-Trbl$^{SLE/G}$, Trbl-HA

G,G. R4-GAL4 driving expression of HA-myr-Trbl resulted in strong membrane-localization of myr-Trbl endogenous nuclear Trbl was low. DAPI overlay in F’. Region in box corresponds to Fig. 5D. Genotype: R4-GAL4>HA-myr-Trbl

H-H”. Ppl-GAL4 driving expression of Flag-Trbl$^{SLE/G}$ detects Trbl accumulation strongly at the cell membrane. DAPI/phallodin overlay in H’. Genotype: Ppl-GAL4>Flag-Trbl$^{SLE/G}$

Development • Supplementary information

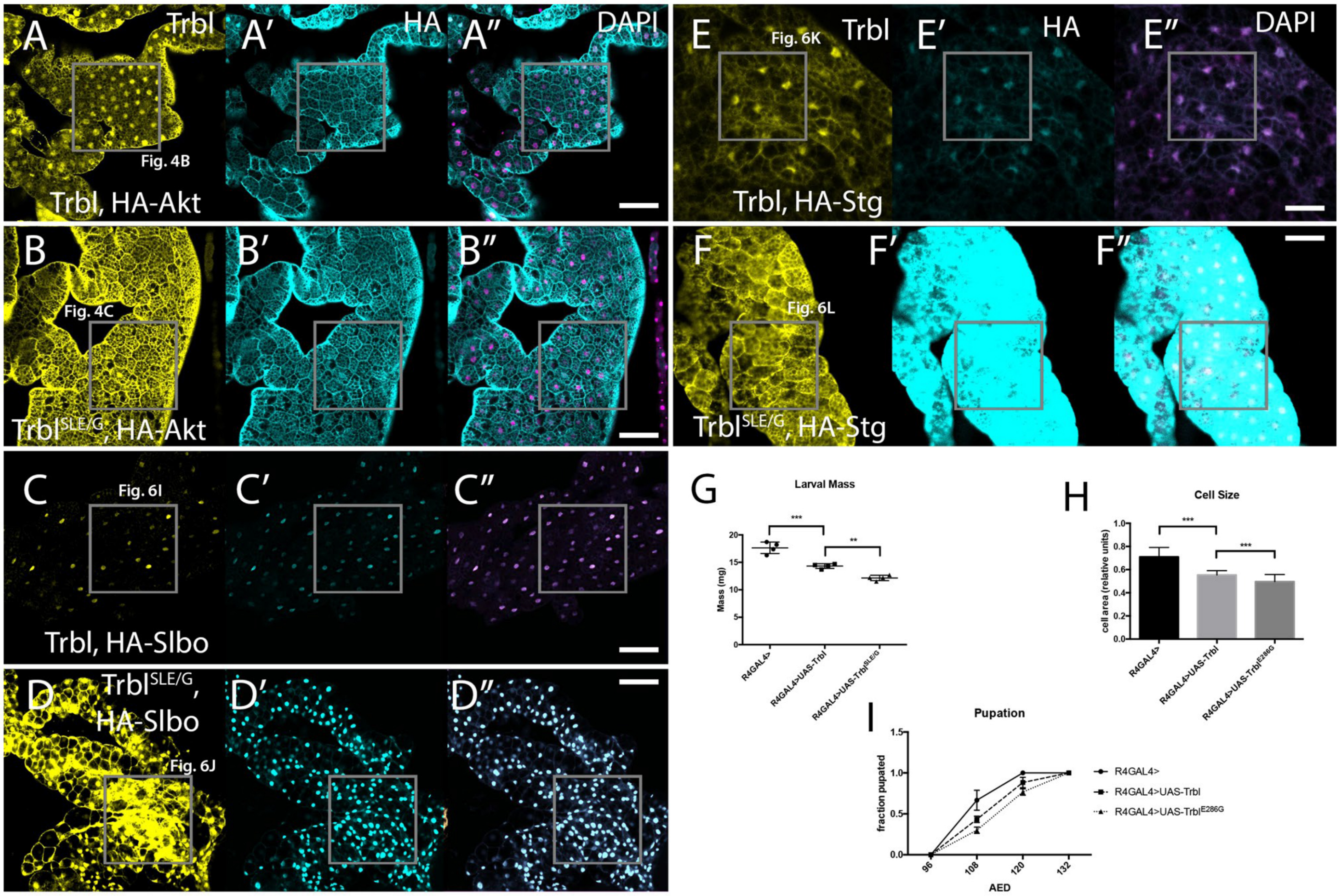

Fig. S5. Trbl$^{SLE/G}$ has dominant negative effects on Trbl targets

A-A”. R4-GAL4 co-expression of Trbl and HA-tagged dAkt revealed Trbl protein is in the nucleus (A) and accumulates at lower levels in the cytoplasm and cell membrane while HA-dAkt is strong at the cell membrane (A’). DAPI overlay in A”. Box corresponds to Fig. 4B. Scale bar in this and all panels for Supplemental figures is 100um.

B-B”. R4-GAL4 co-expression of Trbl$^{SLE/G}$ and HA-tagged dAkt showed Trbl protein accumulates at the cell membrane (B) while HA-dAkt remains unchanged in localization and levels at the cell membrane (B’). DAPI overlay in B”. Region in box corresponds to Fig. 4C. Genotype: R4-GAL4>Trbl$^{SLE/G}$, HA-dAkt

Development • Supplementary information

C-C”. R4-GAL4 co-expression of Flag-Trbl and HA-Slbo results in low levels of nuclear HA-Slbo which colocalized with Trbl. DAPI overlay in G”. Region in box corresponds to Fig. 6G. Genotype: R4-GAL4>UAS-Flag-Trbl, UAS-HA-Slbo

D-D”. R4-GAL4 co-expression of Flag-Trbl$^{SLE/G}$ and HA-Slbo results in increased levels of nuclear HA-Slbo and ectopic Flag-Trbl$^{SLE/G}$ in the nucleus (respective arrows in H and H’). Region in box corresponds to Fig. 6J. Genotype: R4-GAL4>UAS-Flag- Trbl$^{SLE/G}$, UAS-HA-Slbo

E-E”. R4-GAL4 co-expression of Flag-Trbl and HA-Stg results in low levels of HA-Stg (I’) that co-localizes with Trbl (G) at the cell membrane and in the nucleus. DAPI overlay in I”. Region in box corresponds to Fig. 6K. Genotype: R4-GAL4>UAS-Flag-Trbl, UAS-HA-Stg

F-F”. R4-GAL4 co-expression of Flag-Trbl$^{SLE/G}$ and HA-Stg results in greatly increased levels of HA-Stg throughout the cell. DAPI overlay in J”. Region in box corresponds to Fig. 6L. Genotype: R4-GAL4> UAS-Trbl$^{SLE/G}$, UAS-HA-Stg

G. Effects of R4-GAL4 expression of Trbl and Flag-Trbl$^{SLE/G}$ on larval mass.

H. Effects of R4-GAL4 expression of Trbl and Flag-Trbl$^{SLE/G}$ on fat body cell size.

I. Effects of R4-GAL4 expression of Trbl and Flag-Trbl$^{SLE/G}$ on timing of pupation.

Development • Supplementary information

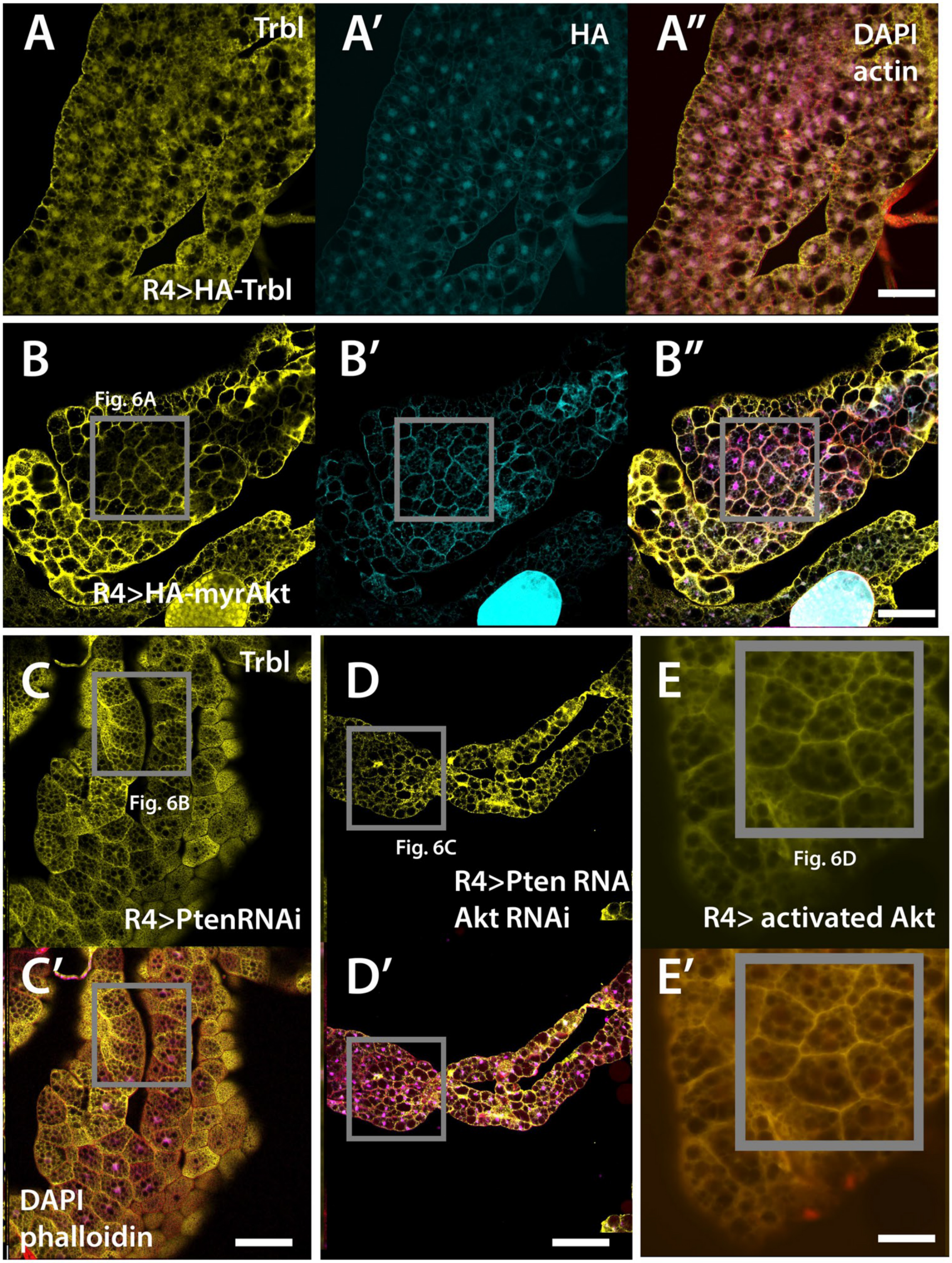
A
Trbl
R4>HA-Trbl
A′
HA
A″
DAPI
actin
B
Fig. 6A
R4>HA-myrAkt
B′
B″
C
Trbl
Fig. 6B
R4>PtenRNAi
C′
DAPI
phalloidin
D
Fig. 6C
R4>Pten RNAi
Akt RNAi
D′
E
Fig. 6D
R4> activated Akt
E′

Fig. S6. Tribbles membrane association in fat body organs: effects of dAkt activation

A-A". R4-GAL4 expression of HA-tagged Trbl revealed endogenous (A) and HA-tagged Trbl protein is stronger in the nucleus (A and A', respectively) and accumulates at lower levels in the cytoplasm and cell membrane. DAPI and phalloidin staining in purple and red, respectively, A"). Genotype: R4-GAL4/UAS-Trbl-HA. Scale bar in this and all panels for Supplemental figures is 100um.

B,B". R4-GAL4 driving HA-Myr-dAkt and UAS-Trbl shows HA-dAkt accumulation at the membrane (B') sufficient to recruit Trbl protein to the cell membrane (B), resulting in low Trbl levels in the nucleus (compare B to A). Genotype: R4GAL4>UAS-Trbl, UAS-HA-Akt. Region in box corresponds to Fig. 2C.

C-C'. R4-GAL4 co-expression of RNAi to Pten and Flag-Trbl results in Trbl accumulation at the cell membrane (C). DAPI overlay in C'. Genotype: UAS-Trbl; R4GAL4/UAS-Pteni. Region in box corresponds to Fig. 2D.

D-D'. R4-GAL4 co-expression of Trbl with RNAi to Pten and RNAi to dAkt results in Trbl accumulation in the nucleus (arrow, E). DAPI overlay in D'. Genotype: UAS-Trbl/UAS-dAkti; R4GAL4/ UAS-Pteni. Region in box corresponds to Fig. 2E.

E,E'. R4-GAL4 expression of UAS-HA-dAktT342D, an GAL4-inducible phosphomimic of Akt, results in endogenous Trbl protein accumulation at the cell membrane (arrow, F). DAPI overlay in E'. Genotype: R4GAL4/ UAS-HA-dAktT342D. Region in box corresponds to Fig. 2F.

Development • Supplementary information

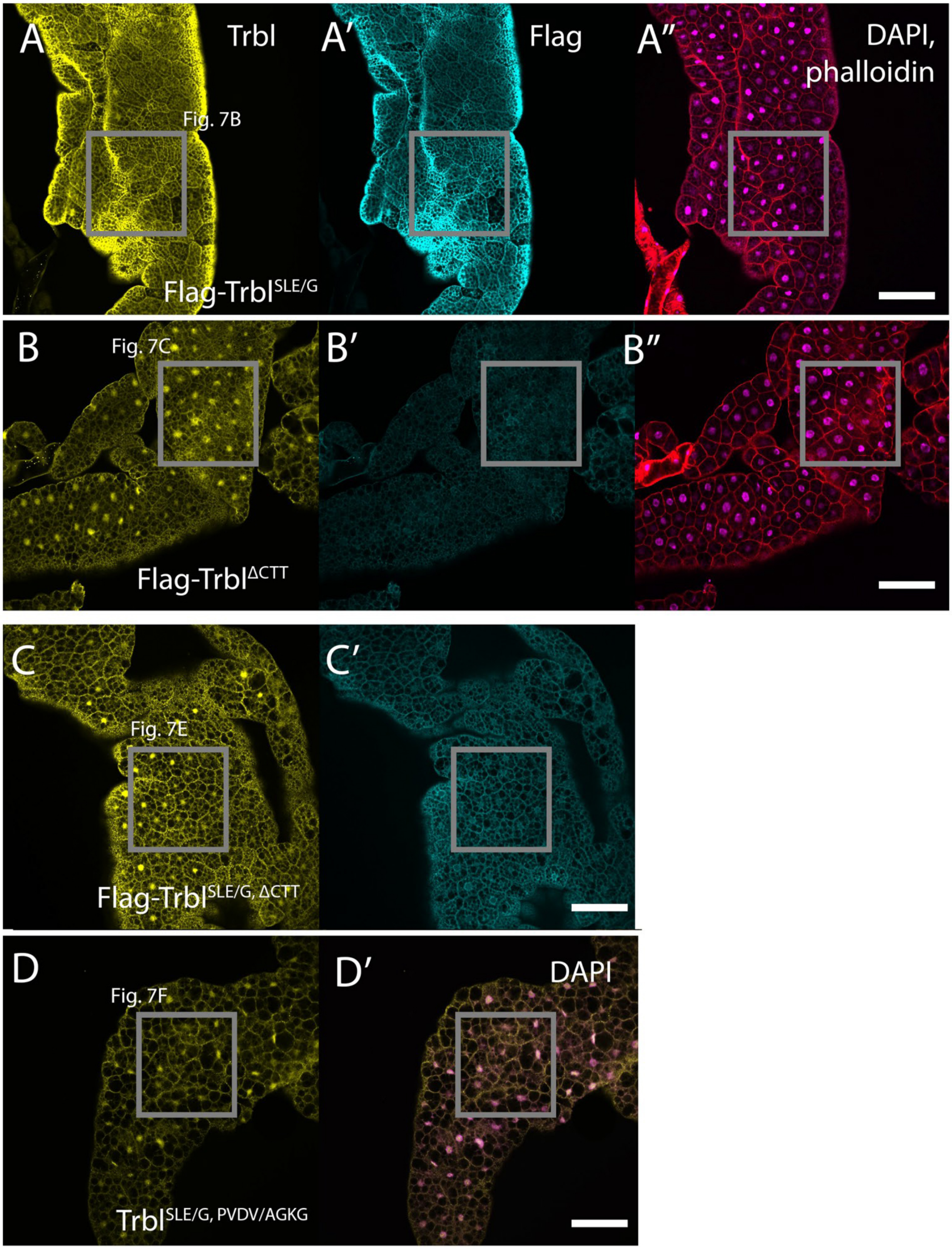

Development • Supplementary information

Fig. S7. C-terminal tail of Trbl is required for Trbl$^{SLE/G}$ phenotypes

A-A". R4-GAL4 expression of Flag-Trbl$^{SLE/G}$ shows Flag accumulation (B') parallels the pattern of aberrant Trbl accumulation at the fat body cell membrane. DAPI and phalloidin overlay in A". Region in box corresponds to Fig. 7B. Genotype: R4-GAL4>Flag-Trbl$^{SLE/G}$. Scale bar in this and all panels for Supplemental figures is 100um.

B-B". R4-GAL4 expression of Flag- Trbl$^{\Delta CTT}$ shows Flag accumulation (C') parallels the pattern of normal Trbl accumulation (C) throughout the cell. DAPI and phalloidin overlay in C". Region in box corresponds to Fig. 7C. Genotype: R4-GAL4>Flag- Trbl$^{\Delta CTT}$

C,C'. R4-GAL4 expression of Flag-Trbl$^{SLE/G,\ \Delta CTT}$ shows Flag (C') accumulates throughout the cell with aberrant punctate staining at the cell membrane. Region in box corresponds to Fig. 7E. Genotype: R4-GAL4 expression of Flag-Trbl$^{SLE/G,\ \Delta CTT}$.

D,D'. R4-GAL4 expression of Flag-Trbl$^{SLE/G,\ PVDV/AGKW}$ shows Trbl accumulation (F) throughout the cell with aberrant punctate staining at the cell membrane. Region in box corresponds to Fig. 7F. Genotype: R4-GAL4>UAS-Flag-Trbl$^{SLE/G,\ PVDV/AGKW}$

Development • Supplementary information

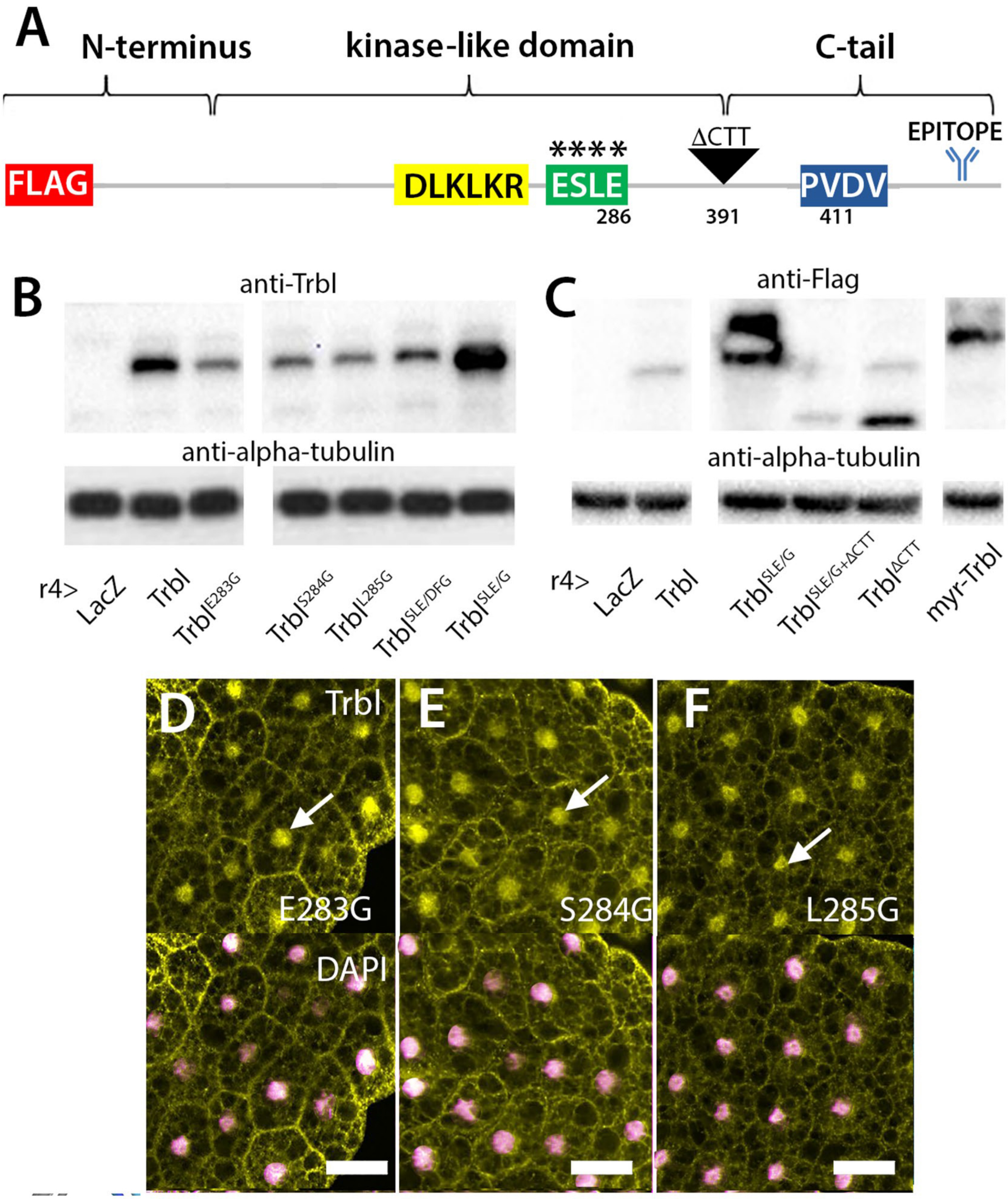

Development • Supplementary information

Fig. S8. C-terminal tail of Trbl is required for Trbl$^{SLE/G}$ phenotypes

A. Map of Trbl protein indicating location of conserved motifs in kinase-like domain and C-terminal tail. Mutations tested in this figure indicated by asterisks. Location of C-tail deletion at residue 391 is indicated and location of Trbl antisera epitope at residue 459 is indicated

B. Western blot analysis of activation loop mutants expressed in larval fat body lysate. Antibodies and genotypes indicated.

C. Western blot analysis of C-terminal tail mutants. Antibodies and genotypes indicated.

D-F. Subcellular distribution of activation loop mutants in larval fat body detected with Trbl antisera in the nucleus (respective arrows). Scale bar in this and all panels is 50um.

D. R4-GAL4 expression of Flag-Trbl E283G shows WT accumulation in the fat body. Genotype: R4-GAL4>UAS-Flag-Trbl E283G

E. R4-GAL4 expression of Flag-Trbl S284G shows WT accumulation in the fat body. Genotype: R4-GAL4>UAS-Flag-Trbl S284G

F. R4-GAL4 expression of Flag-Trbl L285G shows WT accumulation in the fat body. Genotype: R4-GAL4>UAS-Flag-Trbl L285G

Development • Supplementary information

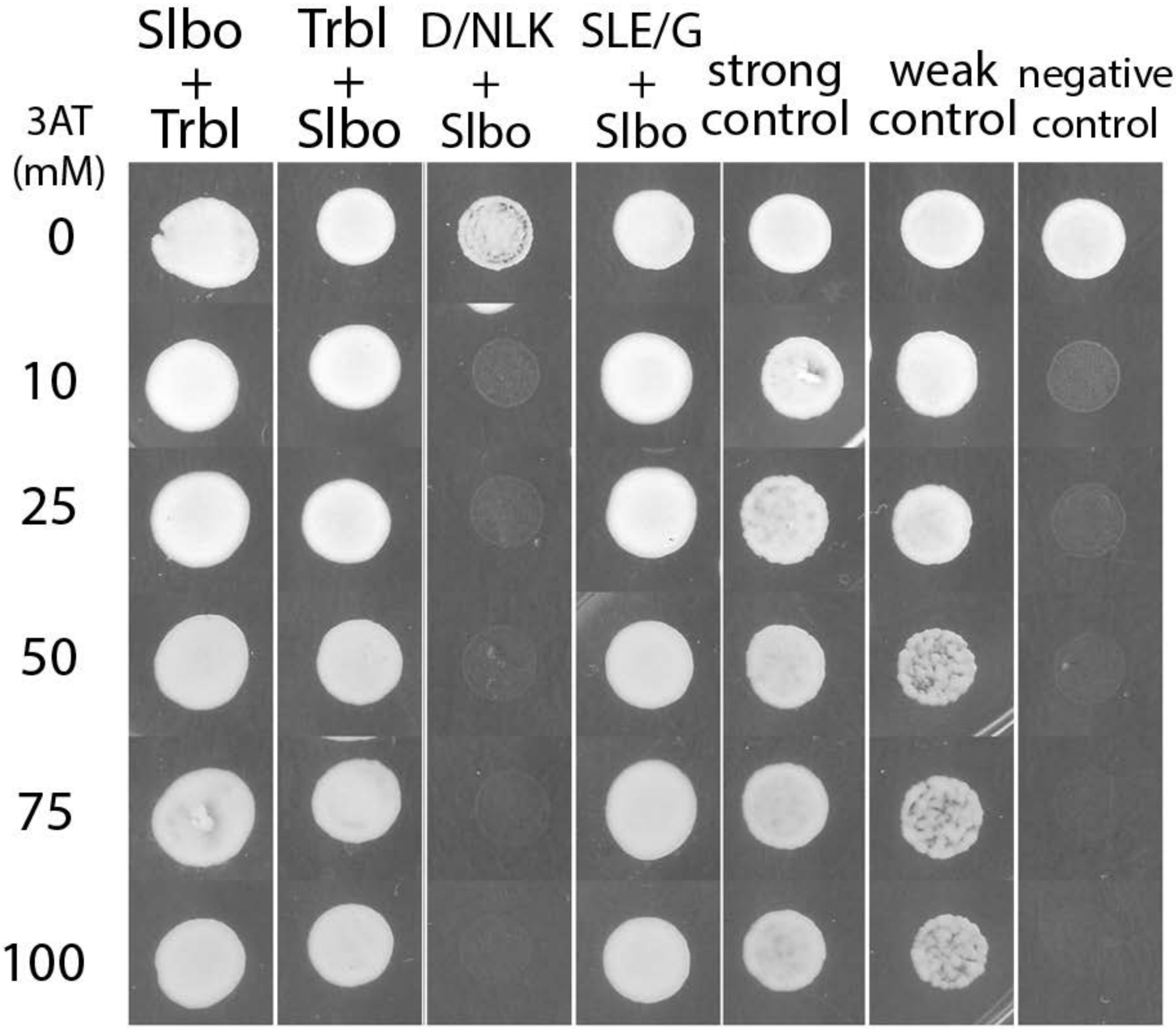

Fig. S9. Trbl$^{SLE/G}$ binds Slbo

Yeast two-hybrid analysis shows binding strength of Trbl-Slbo is unaffected by the Trbl$^{SLE/G}$ mutation whereas the D/NLK mutation disrupts this interaction.

Sequencing of SLE/G

April 1, 2020. In the Dobens lab, we conducted sequencing of both the injection vector used (the plasmid) and genomic DNA from two independent lines of homozygous UAS-E286G flies (using PCR to amplify the transgene) to reveal that both samples carry a mutation in the Tribbles is A856G, which changes the codon from E/G.

```
GCCCCAGGAGAGGGACTCCACGGGTGGGGTGACCGGGGTGTACGAGAACCTGCACACCTA      960
GCCCCAGGAGAGGGACTCCACGGGTGGGGTGACCGGGGTGTACGAGAACCTGCACACCTA      198
GCCCCAGGAGAGGGACTCCACGGGTGGGGTGACCGGGGTGTACGAGAACCTGCACACCTA      201
************************************************************

CATCCGCCACGCGAAGCGACTGTGCGAGACGGAGGCGAGGGCCATATTCCACCAGATCTG      1020
CATCCGCCACGCGAAGCGACTGTGCGAGACGGAGGCGAGGGCCATATTCCACCAGATCTG      258
CATCCGCCACGCGAAGCGACTGTGCGAGACGGAGGCGAGGGCCATATTCCACCAGATCTG      261
************************************************************

TCAGACTGTTCAGGTGTGCCACCGCAACGGGATTATCCTCAGGGACCTCAAGCTCAAGCG      1080
TCAGACTGTTCAGGTGTGCCACCGCAACGGGATTATCCTCAGGGACCTCAAGCTCAAGCG      318
TCAGACTGTTCAGGTGTGCCACCGCAACGGGATTATCCTCAGGGACCTCAAGCTCAAGCG      321
************************************************************

GTTCTACTTCATCGACGAGGCCAGAACGAAACTGCAGTATGAATCACTGGAAGGCTCAAT      1140
GTTCTACTTCATCGACGAGGCCAGAACGAAACTGCAGTATGAATCACTGGGAGGCTCAAT      378
GTTCTACTTCATCGACGAGGCCAGAACGAAACTGCAGTATGAATCACTGGGAGGCTCAAT      381
************************************************** *********

GATCCTCGACGGCGAGGACGATACTCTGAGCGACAAGATCGGTTGCCCACTGTACACCGC      1200
GATCCTCGACGGCGAGGACGATACTCTGAGCGACAAGATCGGTTGCCCACTGTACACCGC      438
GATCCTCGACGGCGAGGACGATACTCTGAGCGACAAGATCGGTTGCCCACTGTACACCGC      441
************************************************************

TCCAGAACTGCTGTGCCCCCAGCAAACGTACAAGGGCAAACCGGCGGACATGTGGTCGCT      1260
TCCAGAACTGCTGTGCCCCCAGCAAACGTACAAGGGCAAACCGGCGGACATGTGGTCGCT      498
TCCAGAACTGCTGTGCCCCCAGCAAACGTACAAGGGCAAACCGGCGGACATGTGGTCGCT      501
************************************************************

GGGCGTGATCCTGTACACCATGTTGGTGGGTCAGTACCCGTTCTACGAGAAGGCCAACTG      1320
GGGCGTGATCCTGTACACCATGTTGGTGGGTCAGTACCCGTTCTACGAGAAGGCCAACTG      558
GGGCGTGATCCTGTACACCATGTTGGTGGGTCAGTACCCGTTCTACGAGAAGGCCAACTG      561
************************************************************
```

March 1, 2021

For fee for service, Genetivision was sent transgenic animals (genotype w: UAS-TrblSLE/G) and reported that they conducted a PCR reaction on fly genomic DNA extract using two primers situated at both ends of your cDNA insert (ATGGATTACAAGGATGACGACGATAAGATGGATA & TCAGCCCATGTCCACATCCGTATCGGGTTC) followed by gel purification of the expected 1.5 kb PCR product. The product was sequenced using the same end primers and a third primer (GGGCACAGCAGTTCTGGAGC) was added in sequencing effort in order to complete the whole region of interest. They reported that the results confirmed the presence of cDNA including the SLE/G point mutation (below).

Development • Supplementary information

Start (0)

Trbl chk1

ATGGATTACAAGGATGACGACGATAAGATGGATA

5' ATGGATTACAAGGATGACGACGATAAGATGGATAACAGTAGCGGT 45

3' TACCTAATGTTCCTACTGCTGCTATTCTACCTATTGTCATCGCCA

1 5

Asp Tyr Lys Asp Asp Asp Asp Lys Met Asp Asn Ser Ser Gly

FLAG (in frame with FLAG)

XmnI

CAAAACAGCAGAACGGCTTCGTCAGCGTCCACAAGCAAAATAGTC 90

GTTTTGTCGTCTTGCCGAAGCAGTCGCAGGTGTTCGTTTTATCAG

Gln Asn Ser Arg Thr Ala Ser Ser Ala Ser Thr Ser Lys Ile Val

(in frame with FLAG)

AACTATTCGTCACCAGTCTCGCCAGGAGTCGCAGCCGCCACCTCC 135

TTGATAAGCAGTGGTCAGAGCGGTCCTCAGCGTCGGCGGTGGAGG

Asn Tyr Ser Ser Pro Val Ser Pro Gly Val Ala Ala Ala Thr Ser

(in frame with FLAG)

TCATCCTCATCATCCAGCAGCAGCGGCATGAGTAGCAGCCAGGAG 180

AGTAGGAGTAGTAGGTCGTCGTCGCCGTACTCATCGTCGGTCCTC

Ser Ser Ser Ser Ser Ser Ser Ser Gly Met Ser Ser Ser Gln Glu

(in frame with FLAG)

GACACCGTACTGGGATTATTTACGCCCAAAAAGGAGTTCCCCAAT 225

CTGTGGCATGACCCTAATAAATGCGGGTTTTTCCTCAAGGGGTTA

Asp Thr Val Leu Gly Leu Phe Thr Pro Lys Lys Glu Phe Pro Asn

(in frame with FLAG)

BsaHI

GCCAAGATGCTGCAGACCATCAGGGAGAAGCTAATGACGCCCGGC 270

CGGTTCTACGACGTCTGGTAGTCCCTCTTCGATTACTGCGGGCCG

Ala Lys Met Leu Gln Thr Ile Arg Glu Lys Leu Met Thr Pro Gly

(in frame with FLAG)

PasI

GGAGCCTGTGACCTTCTGGCCCTGGGCATCGCAGCGGAACCCACA 315

CCTCGGACACTGGAAGACCGGGACCCGTAGCGTCGCCTTGGGTGT

Gly Ala Cys Asp Leu Leu Ala Leu Gly Ile Ala Ala Glu Pro Thr

(in frame with FLAG)

Development • Supplementary information

BclI*

GACCAGCAGCCCGTCAAGCTCATCCAACAGCGTTATCTGATCAGC 360
CTGGTCGTCGGGCAGTTCGAGTAGGTTGTCGCAATAGACTAGTCG
Asp Gln Gln Pro Val Lys Leu Ile Gln Gln Arg Tyr Leu Ile Ser
(in frame with FLAG)

EagI **BsiEI** **BglI**

GCACAGCCTAGTCACATATCGGCCGCCGTGGCTGCCAAGACTCCC 405
CGTGTCGGATCAGTGTATAGCCGGCGGCACCGACGGTTCTGAGGG
Ala Gln Pro Ser His Ile Ser Ala Ala Val Ala Ala Lys Thr Pro
(in frame with FLAG)

GCCTCGTATCGCCACTTGGTCGATCTAACCGCCTCCAATCTGCGC 450
CGGAGCATAGCGGTGAACCAGCTAGATTGGCGGAGGTTAGACGCG
Ala Ser Tyr Arg His Leu Val Asp Leu Thr Ala Ser Asn Leu Arg
(in frame with FLAG)

TGCGTGGACATCTTCACCGGGGAGCAGTTCCTCTGCCGTATTGTA 495
ACGCACCTGTAGAAGTGGCCCCTCGTCAAGGAGACGGCATAACAT
Cys Val Asp Ile Phe Thr Gly Glu Gln Phe Leu Cys Arg Ile Val
(in frame with FLAG)

BstAPI **PvuII**

AACGAACCGTTGCATAAGGTGCAACGAGCCTACTTTCAGCTGCAG 540
TTGCTTGGCAACGTATTCCACGTTGCTCGGATGAAAGTCGACGTC
Asn Glu Pro Leu His Lys Val Gln Arg Ala Tyr Phe Gln Leu Gln
(in frame with FLAG)

MscI

Trbl chk2
GAGCACCATCTATGGCCATCC

CAGCACGACGAGGAGCTACGCCGGAGCACCATCTATGGCCATCCA 585
GTCGTGCTGCTCCTCGATGCGGCCTCGTGGTAGATACCGGTAGGT
Gln His Asp Glu Glu Leu Arg Arg Ser Thr Ile Tyr Gly His Pro
(in frame with FLAG)

ApaLI **EcoRV**

CTGATCCGACCCGTGCACGATATCATACCGCTTACCAAGGATCGC 630
GACTAGGCTGGGCACGTGCTATAGTATGGCGAATGGTTCCTAGCG
Leu Ile Arg Pro Val His Asp Ile Ile Pro Leu Thr Lys Asp Arg
(in frame with FLAG)

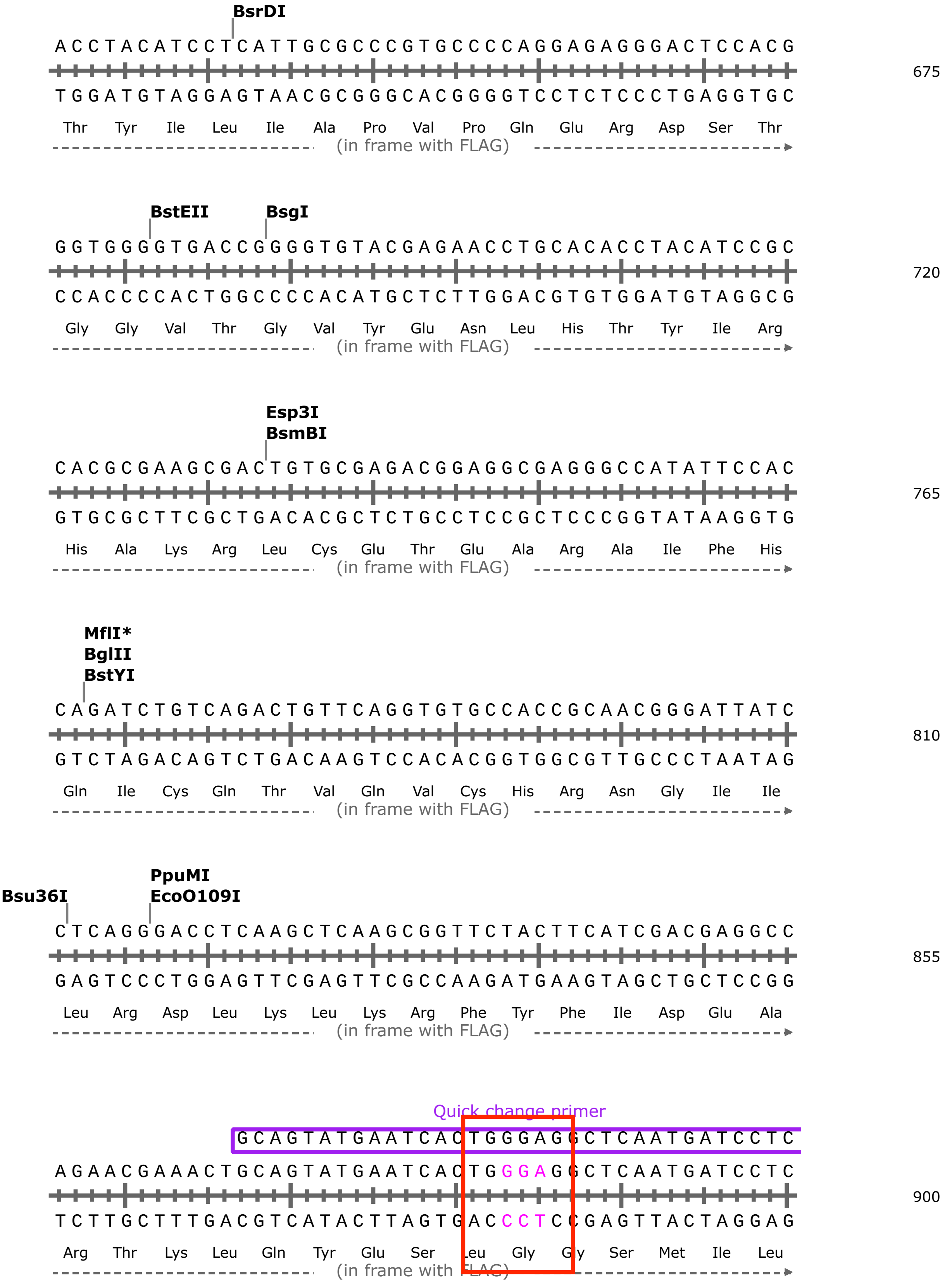
BsrDI
A C C T A C A T C C T C A T T G C G C C C G T G C C C C A G G A G A G G G A C T C C A C G
675
T G G A T G T A G G A G T A A C G C G G G C A C G G G G T C C T C T C C C T G A G G T G C
Thr Tyr Ile Leu Ile Ala Pro Val Pro Gln Glu Arg Asp Ser Thr
(in frame with FLAG)
BstEII
BsgI
G G T G G G G T G A C C G G G G T G T A C G A G A A C C T G C A C A C C T A C A T C C G C
720
C C A C C C C A C T G G C C C C A C A T G C T C T T G G A C G T G T G G A T G T A G G C G
Gly Gly Val Thr Gly Val Tyr Glu Asn Leu His Thr Tyr Ile Arg
(in frame with FLAG)
Esp3I
BsmBI
C A C G C G A A G C G A C T G T G C G A G A C G G A G G C G A G G G C C A T A T T C C A C
765
G T G C G C T T C G C T G A C A C G C T C T G C C T C C G C T C C C G G T A T A A G G T G
His Ala Lys Arg Leu Cys Glu Thr Glu Ala Arg Ala Ile Phe His
(in frame with FLAG)
MflI*
BglII
BstYI
C A G A T C T G T C A G A C T G T T C A G G T G T G C C A C C G C A A C G G G A T T A T C
810
G T C T A G A C A G T C T G A C A A G T C C A C A C G G T G G C G T T G C C C T A A T A G
Gln Ile Cys Gln Thr Val Gln Val Cys His Arg Asn Gly Ile Ile
(in frame with FLAG)
PpuMI
Bsu36I
EcoO109I
C T C A G G G A C C T C A A G C T C A A G C G G T T C T A C T T C A T C G A C G A G G C C
855
G A G T C C C T G G A G T T C G A G T T C G C C A A G A T G A A G T A G C T G C T C C G G
Leu Arg Asp Leu Lys Leu Lys Arg Phe Tyr Phe Ile Asp Glu Ala
(in frame with FLAG)
Quick change primer
G C A G T A T G A A T C A C T G G G A G G C T C A A T G A T C C T C
A G A A C G A A A C T G C A G T A T G A A T C A C T G G G A G G C T C A A T G A T C C T C
900
T C T T G C T T T G A C G T C A T A C T T A G T G A C C C T C C G A G T T A C T A G G A G
Arg Thr Lys Leu Gln Tyr Glu Ser Leu Gly Gly Ser Met Ile Leu
(in frame with FLAG)

Quick change primer
G

GACGGCGAGGACGATACTCTGAGCGACAAGATCGGTTGCCCACTG 945
CTGCCGCTCCTGCTATGAGACTCGCTGTTCTAGCCAACGGGTGAC
Asp Gly Glu Asp Asp Thr Leu Ser Asp Lys Ile Gly Cys Pro Leu
(in frame with FLAG)

BsrBI

TACACCGCTCCAGAACTGCTGTGCCCCCAGCAAACGTACAAGGGC 990
ATGTGGCGAGGTCTTGACGACACGGGGGTCGTTTGCATGTTCCCG
Tyr Thr Ala Pro Glu Leu Leu Cys Pro Gln Gln Thr Tyr Lys Gly
(in frame with FLAG)

CGAGGTCTTGACGACACGGG
Primer 3

BsrFI AhdI

AAACCGGCGGACATGTGGTCGCTGGGCGTGATCCTGTACACCATG 1035
TTTGGCCGCCTGTACACCAGCGACCCGCACTAGGACATGTGGTAC
Lys Pro Ala Asp Met Trp Ser Leu Gly Val Ile Leu Tyr Thr Met
(in frame with FLAG)

BstXI

TTGGTGGGTCAGTACCCGTTCTACGAGAAGGCCAACTGCAACCTC 1080
AACCACCCAGTCATGGGCAAGATGCTCTTCCGGTTGACGTTGGAG
Leu Val Gly Gln Tyr Pro Phe Tyr Glu Lys Ala Asn Cys Asn Leu
(in frame with FLAG)

ATCACCGTCATACGCCACGGGAATGTCCAGATACCCCTGACGCTG 1125
TAGTGGCAGTATGCGGTGCCCTTACAGGTCTATGGGGACTGCGAC
Ile Thr Val Ile Arg His Gly Asn Val Gln Ile Pro Leu Thr Leu
(in frame with FLAG)

TCTAAGTCGGTGCGATGGCTGTTGCTTTCGCTGCTGCGGAAGGAT 1170
AGATTCAGCCACGCTACCGACAACGAAAGCGACGACGCCTTCCTA
Ser Lys Ser Val Arg Trp Leu Leu Leu Ser Leu Leu Arg Lys Asp
(in frame with FLAG)

TACACGGAACGAATGACCGCCAGCCACATCTTCCTTACCCCGTGG 1215
ATGTGCCTTGCTTACTGGCGGTCGGTGTAGAAGGAATGGGGCACC
Tyr Thr Glu Arg Met Thr Ala Ser His Ile Phe Leu Thr Pro Trp
(in frame with FLAG)

Development • Supplementary information

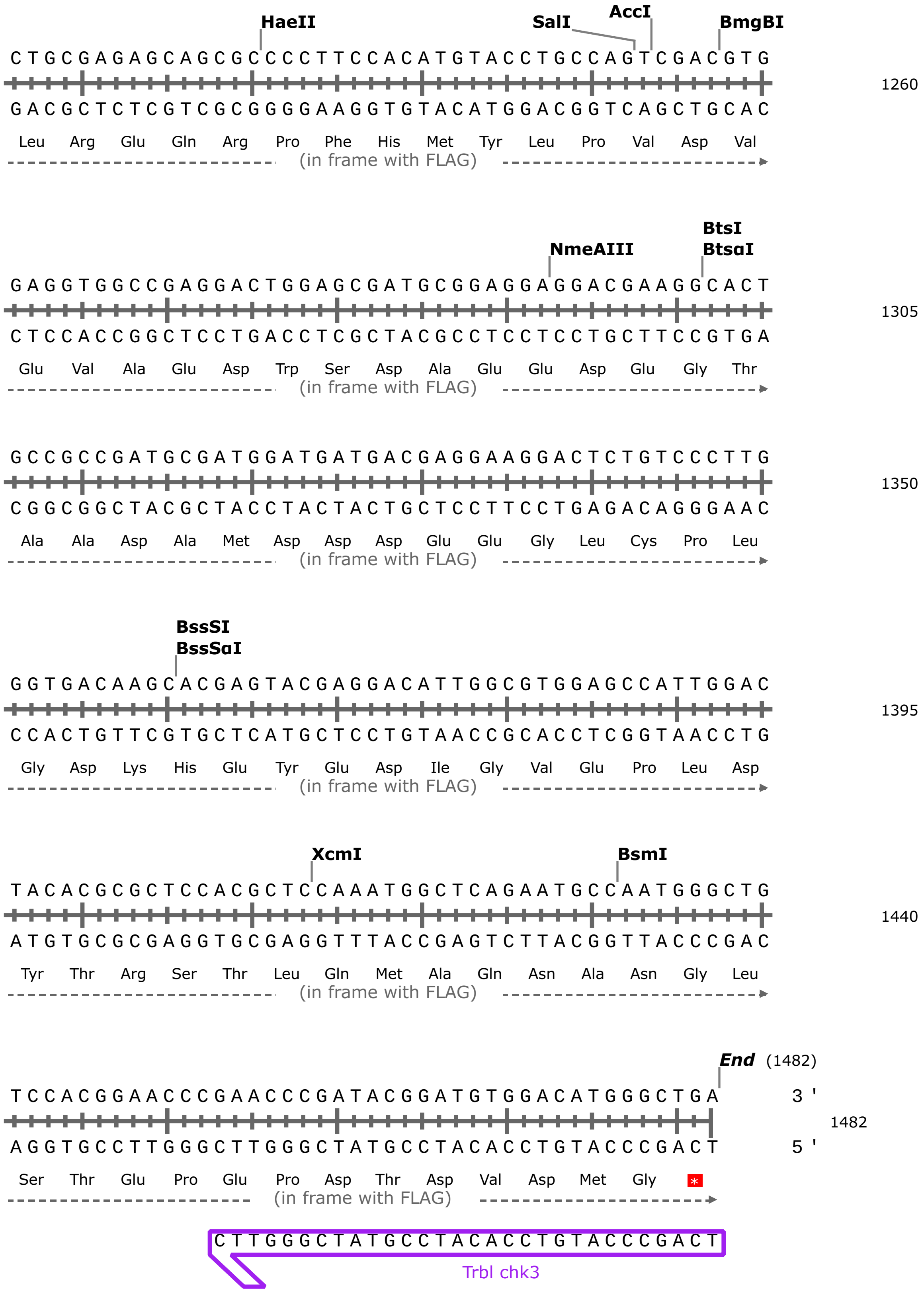

Fig. S10. Sequencing information for Trbl$^{SLE/G}$ mutation

Development • Supplementary information

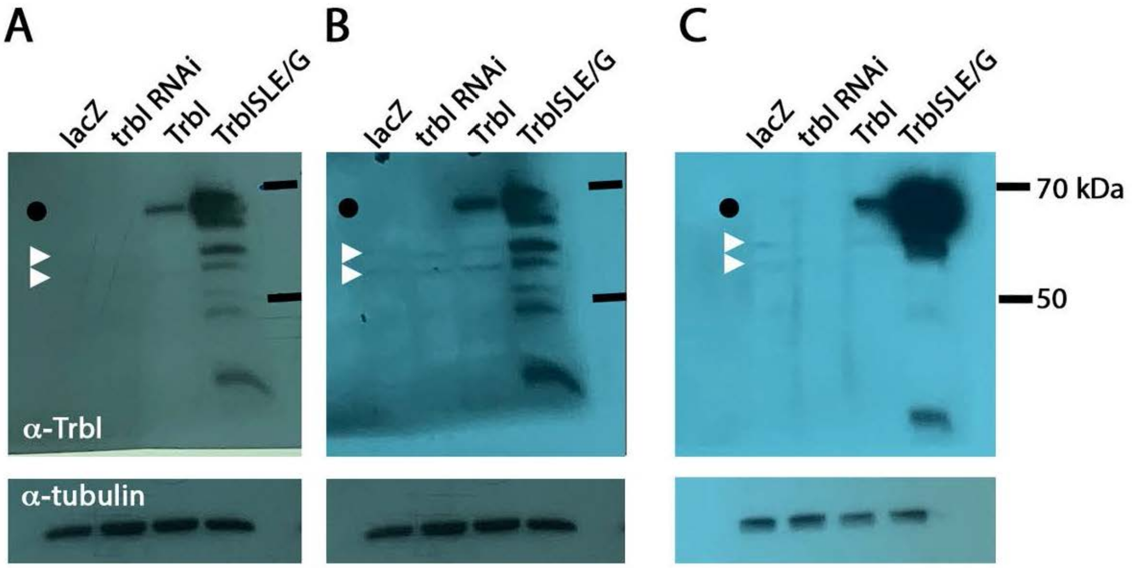

Fig. S11. Western blot of Trbl$^{SLE/G}$

Western blot analysis comparing Trbl levels in fat body lysate from animals expressing lacZ (control), Trbl RNAi, Trbl and Flag-Trbl$^{SLE/G}$ shows higher levels of Flag-epitope tagged Trbl transgenes (black circle) with significantly higher levels of Trbl$^{SLE/G}$ protein levels compared to WT Flag-Trbl expressed under identical conditions from the same attB landing site. In addition, we observe increased stability of proteins of size identical to endogenous Trbl (doublet at approximately 55kDa, white arrowheads) and breakdown products (<50 kDa, detectable by antisera to C-terminal epitope and not visible in control lane). Panels A and B are different exposures of the same experiment and panel C is a separate experiment overexposed to reveal low levels of endogenous Trbl in control lanes.

Development • Supplementary information

Table S1. List of mutations tested

Mutations tested here include: (Column 1) designations made in this work and from previously published work; (column 2) the location and precise amino acids affected; the domain in Trbl affected (column 3); and (columns 3 and 4) the effect on subcellular localization and stability.

designation	mutation	domain affected	localization	stability
WT Trbl			nuclear	low
Myr-FLAG-Trbl		N-terminal myr tag	Nuclear	medium
R148E	R148E	N-lobe	Nuclear	Low
R141Q	R141Q	N-lobe	Nuclear	Low
FLCR/A	R154A	N-lobe	Nuclear	Low
Y167A	Y167A	N-lobe	Nuclear	Low
F168A	F168A	N-lobe	Nuclear	Low
H227A	H227A	pseudokinase	Nuclear	Low
D/NLK	D264N	pseudokinase	Nuclear	Low
DLK/ALA	264DLK/ALA	pseudokinase	Nuclear	Low
LKR/A	R269A	pseudokinase	Nuclear	Low
E283G	E283G	pseudokinase	Cell membrane	High
S284A	S284A	pseudokinase	Nuclear	Low
SLE/G	S284G	pseudokinase	Nuclear	Low
SLE/DFG	284SLE/DFG286	SLE to DFG	Nuclear	Low
SLE/DFG FLCR/VAIK	151FLCR/VAIK154 284SLE/DFG286	pseudokinase	Nuclear	Low
L285G	L285G	pseudokinase	Nuclear	Low
DE391trunc	391TAG	MEK1 truncated	Nuclear	Low
SLE/G delta MEK	E283G-391TAG	SLE/G MEK1 truncated	Nuclear	Low
MEK1	396W/A	MEK1 motif	Nuclear	Low
PVDV	408PVDV/AGKW411	C-tail motif mutant	Nuclear	Low
SLE/G,PVDV	E283G-408PVDV/AGKW411	SLE/G, C-tail motif mutant	Nuclear	Low
DE409trunc	409TAG	COP1 deletion	Nuclear	Low
DE426trunc	426TAG	COP1 deletion	Nuclear	Low
COP1	436E/G	COP1 motif	Nuclear	Low
