# Supplementary material for: The Drosophila pseudokinase Tribbles translocates to the fat body membrane in response to fasting to modulate insulin sensitivity
Source: Development. 2025 Apr 28;152(8):dev204493. doi: 10.1242/dev.204493 (PMC12070071; doi:10.1242/dev.204493)
Supplement: Supplementary file 1 [file develop-152-204493_review_history.pdf]

The *Drosophila* pseudokinase Tribbles translocates to the fat body membrane in response to fasting to modulate insulin sensitivity

Zachary Fischer, Christopher Nauman, Shima Shayestehpour, Laramie Pence, Samuel Bouyain, Xiaolan Yao and Leonard Dobens

DOI: 10.1242/dev.204493

Editor: Irene Miguel-Aliaga

Review timeline

Original submission:	7 April 2023
Editorial decision:	14 June 2023
Resubmission:	15 November 2024
Editorial decision:	31 January 2025
First revision received:	19 February 2025
Accepted:	27 February 2025

Original submission

First decision letter

DEVELOP/2023/201841

MS TITLE: Trafficking of the Tribbles pseudokinase to the membrane regulates insulin sensitivity in the Drosophila fat body

Authors: Zachary Fischer, Christopher Nauman, Laramie Pence, Samuel Bouyain, Xiaolan Yao and Leonard Dobens
Dear Dr. Dobens,

I have now received all the referees reports on the above manuscript, and have reached a decision. The referees' comments are appended below, or you can access them online: please go to BenchPress and click on the 'Manuscripts with Decisions' queue in the Author Area.

As you will see from their reports, the referees recognise the potential of your work, but they also raise significant concerns about it - particularly those concerning data analysis, data presentation and possible effects of the inserted tag.. Given the nature of these concerns, I am afraid I have little choice other than to reject the paper at this stage.

However, having evaluated the paper, I do recognise the potential importance of this work. I would therefore be prepared to consider as a new submission an extension of this study that contains new experiments, data and discussions and that address fully the major concerns of the referees. The work required goes beyond a standard revision of the paper. Please bear in mind that the referees (who may be different from the present reviewers) will assess the novelty of your work in the context of all previous publications, including those published between now and the time of resubmission.

© 2025. Published by The Company of Biologists under the terms of the Creative Commons Attribution License (https://creativecommons.org/licenses/by/4.0/).

Previous Reviews

Reviewer 1 Advance Summary and Potential Significance to Field:
The paper by Fischer and colleagues tells a clear story of potential importance to the Trbl/AKT fields. Focused on a genetically tractable insulin signaling AKT/Trbl paradigm (the fat body) the paper builds on previous work evaluating how the Trbl pseudokinase (TRIB1-3 in vertebrates) interferes with AKT signaling. A combination of elegant genetics and microscopy is combined with immunoblotting. In flies, Trb blocks Akt signaling through direct binding, and during fasting or IR engagement (or expression of constitutively active AKT, ie as a function of Akt activity), Trb is observed to relocate from a predominantly nuclear site to the cell membrane. Mechanistically, this blocks AKT activity (and downstream effectors). Exogenous Trbl also appears to target endogenous Trbl, similar to multimerization established previously (and in human TRIB2 via a structure). Mutation in the Trbl gene encoding key conserved residues have little effect, except for one, in which the second Glu of the ESLE motif is converted to Gly, which is 1) hyperstable, 2) driven to the membrane at the expense of nuclear Trbl, 3) drives enhanced interaction with Akt and 4) also relevant to Trbl multimerization. SLE/G Trbl works in a dominant negative manner, in a fashion that involves the C-tail, a known effector for conformational switching in vertebrate Tribbles.

Reviewer 1 Comments for the Author:
For revision, I encourage only minor changes. The work is robust, and very well controlled throughout, including painstaking analysis of the reliability and relevance of the SLE/G mutation.

Minor points:
1) p.2 Abstract talks about mutagenesis of the Trbl central kinase domain. This should be central 'pseudokinase' domain.
2) A citation to PMID: 23863165 early on will help readers understand the pseudokinase field a bit better, given the discussions of motifs (Figure 3A) and 'activation loop'. In this context, worth explaining that the activation segment is defined in kinases and pseudokinases as the region between DFG/SLE and APE, rather than SLE being 'in' the activation loop. The discussion takes care of some of this, but worth emphasising early on.
p. 3. 'although kinase activity has been reported' should be replaced with 'although very low vestigial kinase activity has been reported'
3) p. 6. the catalytic DLK motif should be the 'catalytic' DLK motif.
4) p. 8. I would tone down the title, which talks about activation loop being required; the SLE/G mutation does not ablate the loop, just likely changes the conformation of Trbl.
5) The alphafold analysis of Drosophila Trbl pseudokinase domain should be included in this paper, not just a model of a few amino acids. This provides an opportunity to compare the Trbl prediction with the known TRIB1 and TRIB2 architectures, which will increase the relevance of the work. AF2 is well known to bias predictions to 'active-like' conformations of kinases.
6) Figure 7B/C are not particularly convincing; markers should be added, and the full blots shown. Levels of 'DFG', and especially E283G/S284G look like they go down (increased turnover). I think this is worth discussing.

Reviewer 2 Advance Summary and Potential Significance to Field:
In this paper Fischer et al. investigate the Drosophila Tribbles pseudokinase, which has been shown to interact with Akt and block its activity to regulate the insulin signaling pathway. They assess changes in the subcellular localization of Tribbles in the fat body using antisera and tagged proteins. The authors’ premise is that Tribbles is normally found in the nucleus but accumulates at the membrane in response to fasting or increases in Akt activity. They identify a single amino acid in a conserved region of the protein that they suggest is required for Tribbles turnover and release from

© 2025. Published by The Company of Biologists under the terms of the Creative Commons Attribution License (https://creativecommons.org/licenses/by/4.0/).

the plasma membrane. Further, the C-terminal tail of Tribbles is proposed to mediate homo-dimerization.

Reviewer 2 Comments for the Author:
The change in subcellularization of Tribbles in response to dietary stress is an interesting observation, suggesting context dependent roles for this pseudokinase. The manuscript builds on previous work demonstrating that Tribbles binds and inhibits Akt and consequently regulates the insulin signaling pathway.
This has the potential to be an interesting story, however there are significant problems with the present version of the paper. These include, but are not an exhaustive list:

1.No scale bars are shown in any of the figures and need to be added to every panel.

2.Monochrome would be clearer for single label experiments, and double labelled images should be merged to show co-localization. An antibody labeling the nuclear lamina would have helped to demarcate the nuclei.

3.There are five instances of “data not shown”.

4.The images are not of uniform quality.

5.Does Tribbles interact with Cdc42? Previous papers suggest that TRIB and Tribbles interact with Cdc25 (not Cdc42) in human (Liang et al. 2016) and Drosophila (Mata et al., 2000; Grosshans and Wieschaus, 2000; Seher and Leptin, 2000). The references in the first paragraph of the manuscript should be corrected and not refer to Grosshans and Wieschaus, 2001, which is a meeting abstract.

6.The Tribbles-CRISPR-GFP construction is not described in the paper, but is refenced to a paper in preparation.

7.The HA-tag is at the C-terminus of Tribbles. The authors state that the C-terminus is required for interactions mediated by the activation loop. Might the tag interfere with Tribbles function? The GFP tag in the Tribbles-CRISPR-GFP is also presumably C-terminal, although the fusion is not described in the paper (it should be).

8.The authors attempt to quantify levels of fluorescence in several figures. It might have been more useful to state how many of the 90 cells scored in each fat body had nuclear Tribbles localization and how many had membrane localization (or a mixture of each).

9.The nuclear staining is not always uniform, example figure 3C compared to 3A. In 3C the staining looks like the periphery of the nucleus. This is not discussed.

10.The hours in the text don’t always correspond with the figures, for example bottom of page 5, figure 3E.

11.Mislabeling in the text - 2D should be 3D, 2F should be 3F.

12.Figure 3 legend (A, A’) references B instead of A’.

13.What are bright spots in figure 5G?

14.Add the reference for Tribbles forming homo dimers – bottom of page 7.

© 2025. Published by The Company of Biologists under the terms of the Creative Commons Attribution License (https://creativecommons.org/licenses/by/4.0/).

15.String is a Cdc25 homologue, not Cdc42 (page 9).

16.Page 10 “Trib LKR/A localized strongly to the cell membrane in half of the fat body organs examined (n=10)”. Figure 8I – the membrane staining looks weaker rather than stronger. What happens in the other 50% of the fat bodies?

17.Page 12, discussion – mutation of R269, predicted to interact with E286, “localized strongly” to the cell membrane in fed larvae, Figure 8I – see above.

Reviewer 3 Advance Summary and Potential Significance to Field:
In this MS, Fischer and cols study the cellular localization of Trbl and the functional implications by using the Drosophila fat body as a model. They describe different changes in cellular localization after different genetic manipulations and dietary changes. They connect their findings with changes in insulin sensitivity.

The topic analyzed is interesting. However, the MS in its current version is very preliminary and not ready for publication.

Reviewer 3 Comments for the Author:
General comments:

The authors claim changes in the cellular distribution of different proteins and provide examples but changes in intensity in the different cell parts are not quantified. To show their results in a convincing manner changes in the intensity of the different markers between the different parts of the cells (nucleus, cytoplasm, and membrane) should be quantified and statistical analyses should be provided. Besides, markers as phalloidin and DAPI should be used in all their immunostainings to mark those cellular compartments.
The immunostainings shown should include scale bars.
Some of the analyses lack obvious controls. See some examples in the specific comments presented below.

Specific comments, Fig by Fig, can be found below:

Fig 2: markers for nuclei and membrane should be shown, together with HA and Trbl .
Quantifications of membrane and nucleus signal in the genetic context analyzed are required.

Fig 2A shows dAkt over-expressing fat bodies. Trbl protein should be shown in wild type organs. This control is required to compared with the fat bodies where dAkt (2A) and Akt+Trbl (2B) are upregulated.

Fig 2A and B. In the text, the authors mentioned that “Trbl antisera detected accumulation of endogenous Trbl or a Flag-tagged Trbl transgene predominantly in the fat body cell nucleus (respective arrows in 2A and 2B) and weakly in the cytoplasm and at the cell membrane.” It would be helpful if the Figs shows labels for both, Trbl or a Flag-tagged Trbl.
In the Fig, it labels only “Trbl”. The authors claim it accumulates in the nucleus. A nucleus marker (eg, DAPI) should be used to label the nucleus. The Trbl signal between A and B looks quite different. Why is that? This should be clarified and discussed.

The authors stated, “As shown in Fig. 2, Trbl antisera detected accumulation of endogenous Trbl or a Flag-tagged Trbl transgene predominantly in the fat body cell nucleus (respective arrows in 2A and

© 2025. Published by The Company of Biologists under the terms of the Creative Commons Attribution License (https://creativecommons.org/licenses/by/4.0/).

2B) and weakly in the cytoplasm and at the cell membrane." They however do not mention that that corresponds to tissues over-expressing dAkt. This should be mentioned. Otherwise, it is confusing and difficult to interpret. In the next sentence they claim: "Co-expression of an HA-dAkt showed accumulation of dAkt predominantly at the cell membrane (Fig. 2A' and 2B'; Bischof et al., 2013)." Co-expression with what? Based on the Fig, it seems they meant Trbl but this should be clearly explained in the text.

I find this first part of the results confusing and should be revised to facilitate the understanding by the reader.

The authors claim that r4>HA-myr-dAkt "localized strongly to the fat body cell membrane". Cell membrane markers should be used to mark positively the cell membrane. Besides, it seems HA might be in the membrane, but it is also present in other cytoplasmic regions. To claim that it is enriched in the membrane this signal should be quantified and compared with other cellular structures. The same comment applies to the Trbl signal.

Trbl levels look way stronger in Fig 2C, D than 2A, B. Is the gene expressed at higher levels or protein stability is affected? mRNA and protein quantification should be provided to clarify this important issue.

Fig 2E should show the quantification in wild type and Trbl(only)-expressing animals. These controls are required.

Fig 2G should be compared with fat bodies where PTEN-RNAi is expressed in the absence of Trbl upregulation. Important control missing.

Fig 2H is missing Trbl protein in dAkt-RNAi fat bodies with and without Tribl overexpression.
-

Fig 3A-E. Is that Trbl increase caused by an increase in gene expression? mRNA levels should be quantified and provided.

The authors claim that "we found increased Trbl protein accumulation at the membrane (arrows, Fig. D and F, respectively) and corresponding reduced levels in the nucleus." The Fig number should be indicated – it is missing. Besides, the nuclear levels, according to the quantification below, are not reduced but increased (see Fig 3F´´). I assume the first D´´ should read C´´.
-

Fig 4:

The authors claim: "Endogenous Trbl and WT Flag-Trbl localized predominantly in the nucleus of fat body cells of well-fed animals (arrows, Fig. 4B and C, respectively), with lower levels in the cytoplasm and levels weakly detectable at the cell membrane." I can´t see that in those panels. Nuclear and membrane markers (phalloidin and DAPI, for example) are required to label those cellular structures.

"When stained with Trbl antisera, we detected Flag-TrblFLCR/A and Flag-TrblD/NLK mutations with a distribution similar to WT (arrows mark nucleus, Fig. 4D and 4E respectively)." I disagree. The Trbl pattern in D and E is obviously different.

"In contrast, the Flag- TrblSLE/G mutation resulted in strong accumulation of Trbl at the cell membrane (arrow, Fig. 4F) with comparatively reduced nuclear and cytoplasmic levels, a distribution

© 2025. Published by The Company of Biologists under the terms of the Creative Commons Attribution License (https://creativecommons.org/licenses/by/4.0/).

profile confirmed by co-localization with phalloidin staining in optical cross-sections (Fig. 4F')." Trbl levels in 4F look higher. Not only in the membrane but all over the cell. Is it a matter of localization or protein stability os also affected.
Protein quantification needs to be provided to answer this central question.
-

Fig 5:

Nuclear and membrane markers (phalloidin and DAPI, for example) are required.

I can´t see in the examples provided what the authors claim in the text. Signal quantification in the different cellular structures analyzed as well as statistical analysis will be required to show those results in a convincing manner.
-
Fig 6I: the 65 kDa mark in the Trbl-SLE/G is way higher in intensity than the band in the Trbl sample. The authors claim that is 6 times higher. In the western it seems to be way higher. How was that quantification done?

How is the expression of both transgenes? To claim that the difference in levels is due to protein turnover the authors should show the mRNA levels in both conditions.
-
Fig 7

Nuclei should be labeled and shown in Fig 7F.

Fig 7H, I: Although the nuclear signal is higher, it is also the background. Those images seem to be captured using different settings. The use of clones could help to show these results in a more convincing manner.
Quantifications and statistical analysis of the signal in the different cellular compartments, as in the rest of the MS, will also be required.

First Resubmission

<u>Author response to reviewers</u>

<u>**Point-by-point reply to reviewers**</u>

We have made three significant changes adding new data acquired by us, guided by new data published by others, and in response to the original set of reviewers' comments. These are:

a) Every figure has been updated to include nuclear and cell membrane marker overlays requested by reviewers. Supplemental data for each updated figure shows the corresponding entire fat body organ and is included to convey the effect of genetic manipulations on tissue structure.
(please note that reduction in figure quality occurred during the upload process - it was necessary to reduce figure size and to save images with reduced quality to achieve proper manuscript size - the original figures of high quality are available)

b) Two new pieces of experimental data have been added to augment the model that Trbl gene expression increases following reduced insulin signaling (Fig. 1C) and that endogenous Trbl protein increases at the membrane following increased dAkt

© 2025. Published by The Company of Biologists under the terms of the Creative Commons Attribution License (https://creativecommons.org/licenses/by/4.0/).

activity (Fig. 6D).

c) The order of the figures has been changed to present the data with a more logical flow (Fig. 2 moved to Fig. 6 and 3-6 drop in number) and the discussion presents a more straightforward and coherent explanation of the results based on the new data cited and newly published data by labs studying mammalian Trbl proteins.

Using the compare function of Word to compare the revised text to the original submission (attached as a word file) reveals extensive editing that accommodates the changes requested by reviewers listed below.

Reviewer #1 acknowledges the strengths of the manuscript, noting "clear story of potential importance to the Trbl/AKT fields" and concludes "the work is robust, and very well controlled throughout, including painstaking analysis of the reliability and relevance of the SLE/G mutation" This reviewer asks for several changes to the manuscript (in **bold**) which we have responded to as follows:

1) A citation to PMID: 23863165 early on will help readers understand the pseudokinase field a bit better, given the discussions of motifs (Figure 3A) and 'activation loop'. In this context, worth explaining that the activation segment is defined in kinases and pseudokinases as the region between DFG/SLE and APE, rather than SLE being 'in' the activation loop. The discussion takes care of some of this, but worth emphasising early on.
Citations have been updated throughout – that is evident in the highlighted comparison of the two versions – Eyers et al 2017 Fig. 3 refers to this SLE motif as within the activation loop so we keep it here.

2) The alphafold analysis of Drosophila Trbl pseudokinase domain should be included in this paper, not just a model of a few amino acids. This provides an opportunity to compare the Trbl prediction with the known TRIB1 and TRIB2 architectures, which will increase the relevance of the work. AF2 is well known to bias predictions to 'active-like' conformations of kinases.
Alphafold analysis has been removed from the revised manuscript.

3) Figure 7B/C are not particularly convincing; markers should be added, and the full blots shown. Levels of 'DFG', and especially E283G/S284G look like they go down (increased turnover). I think this is worth discussing
The strong increase in stability of SLE/G was not recapitulated in any other activation loop mutation and we do not see any decreases in levels or connected changes in localization of the other activation loop mutations in Supplemental Fig 8B and 8E-G. All mutations summarized in (new) Table 1.

4) p.2 Abstract talks about mutagenesis of the Trbl central kinase domain. This should be central 'pseudokinase' domain.
We make this change

5) p. 3. 'although kinase activity has been reported' should be replaced with 'although very low vestigial kinase activity has been reported'
We will call it "low" – this is a contentious thing among biochemists and structural biologists studying the kinase strength of the Trib2 protein and not important to the manuscript

3) p. 6. the catalytic DLK motif should be the 'catalytic' DLK motif.
Revised to clarify

4) p. 8. I would tone down the title, which talks about activation loop being required; the SLE/G
mutation does not ablate the loop, just likely changes the conformation of Trbl.
In this revision we change the title and hope the reviewer likes the update

© 2025. Published by The Company of Biologists under the terms of the Creative Commons Attribution License (https://creativecommons.org/licenses/by/4.0/).

Reviewer #2 agrees “change in subcellularization of Tribbles in response to dietary stress is an interesting observation, suggesting context dependent roles for this pseudokinase…[with] the potential to be an interesting story…” Reviewer #2 has the following criticisms in **bold** that we address:

1) No scale bars are shown in any of the figures and need to be added to every panel.
Proper scale bars have been added to all figures and supplementary figures

2) Monochrome would be clearer for single label experiments, and double labelled images should be merged to show co-localization. An antibody labeling the nuclear lamina would have helped to demarcate the nuclei.

The cell types are large and rather simple, and our color choices used throughout the manuscript are consistent: yellow to indicate Trbl protein, magenta for DAPI, red for phalloidin, blue for HA and green for GFP. We mark cell membrane and nucleus where relevant. Thus, we think monochrome will give up the strength of that simple coding. Overlays have been introduced throughout.

3) There are five instances of “data not shown”

The revision has removed several instances of data not shown by adding the data (each Supplemental figure includes the entire organ now) or by removing a mention of it if it is not relevant. This revision retains four instances of “data not shown” in this version, all of which are fairly trivial but we can add if reviewers seek this data:

1- p6: Sequencing the SLE/G mutation to ensure a second site modifier is data not shown.
Fee-for- service sequencing data available on request.
2- p7: An overexposed blot discussed on p7 where endogenous Trbl can be detected is data not shown.
Available data if needed.
4- p9: Lethality of Trbl-GAL4 driving SLE/G is presented as data not shown on p9. *Available data if needed.*
5- p13 Lethality of the GFP knock-in allele in material and methodsis data not shown on p13.
Available data if needed.

4) The images are not of uniform quality

In the process to upload the figures, we had to go back and reduce the quality of some pdfs to get under a file size limit – in this revision we will try to maintain figure quality throughout.
In this revision we have struggled also to upload quality images within the 20mB size limit. Higher quality images are available.

5) Does Tribbles interact with Cdc42? Previous papers suggest that TRIB and Tribbles interact with Cdc25 (not Cdc42) in human (Liang et al. 2016) and Drosophila (Mata et al., 2000; Grosshans and Wieschaus, 2000; Seher and Leptin, 2000). The references in the first paragraph of the manuscript should be corrected and not refer to Grosshans and Wieschaus, 2001, which is a meeting abstract.

We fix these typos and thank the reviewer for catching them. The references have been updated throughout.

6) The Tribbles-CRISPR-GFP construction is not described in the paper, but is referenced to a paper in preparation

Based on the confusion regarding the nature of this tool (below) we have decided to describe its construction in detail in the Materials and Methods – thus this manuscript will be the reference for this Crispr Trbl GFP knock-in tool going forward. This will clear up the confusion Reviewer #2

© 2025. Published by The Company of Biologists under the terms of the Creative Commons Attribution License (https://creativecommons.org/licenses/by/4.0/).

had in the next bullet point.

7. The HA-tag is at the C-terminus of Tribbles. The authors state that the C-terminus is required for interactions mediated by the activation loop. Might the tag interfere with Tribbles function? The GFP tag in the Tribbles-CRISPR-GFP is also presumably C-terminal, although the fusion is not described in the paper (it should be)

This revised manuscript includes a detailed description of the Trbl-GFP Crispr allele in materials and methods and it is worth noting that it is a GFP knock into exon 1 with multiple stop codons so it is not a tagged protein. We hope this resolves confusion about this tool.

We use the HA tag to distinguish endogenous Trbl from SLE/G-HA or Trbl-HA. The C-terminal tag DOES interfere with Trbl function in protein in protein turnover as the reviewer surmises. However, that fact is not relevant to our focus on dAkt activation and our use of the HA tag as a way to follow around SLE/G-HA localization in the cell. We can respond to this at greater length if necessary.

8. The authors attempt to quantify levels of fluorescence in several figures. It might have been more useful to state how many of the 90 cells scored in each fat body had nuclear Tribbles localization and how many had membrane localization (or a mixture of each).

We think the approach presented in Fig. 1 and Fig. 2 to measure fluorescence averaged across 90 cell bodies in three independent experiments makes the point clearly – because fat body cells were of different sizes, there were problems aligning the edge of some cells but the overall presentation shows the effect clearly .

9. The nuclear staining is not always uniform, example figure 3C compared to 3A. In 3C the staining looks like the periphery of the nucleus. This is not discussed.

This is a good point and we note this on p4 now. Also, the addition of nuclear and membrane stains throughout addresses this, too.

10. The hours in the text don't always correspond with the figures, for example bottom of page 5, figure 3E.

Figures revised and we have been very careful about labeling.

11. Mislabeling in the text - 2D should be 3D, 2F should be 3F.
Figures revised and we have been very careful about labeling.

12. Figure 3 legend (A, A') references B instead of A'.
Figures revised and we have been very careful about labeling.

13. What are bright spots in figure 5G?
Trbl accumulation is punctate at the membrane in cells expressing SLE/G double mutants and this is now mentioned on p10

14. Add the reference for Tribbles forming homo dimers – bottom of page 7
References have been carefully updated.

15. String is a Cdc25 homologue, not Cdc42 (page 9).
We fix this throughout

16. Page 10 "Trib LKR/A localized strongly to the cell membrane in half of the fat body organs examined (n=10)". Figure 8I – the membrane staining looks weaker rather than stronger. What happens in the other 50% of the fat bodies?

We have examined this TrblLKR/A mutant more carefully in this revision and have determined

© 2025. Published by The Company of Biologists under the terms of the Creative Commons Attribution License (https://creativecommons.org/licenses/by/4.0/).

that it does not localize to the cell membrane like SLE/G so this information is removed (update added to Table 1)

17. Page 12, discussion – mutation of R269, predicted to interact with E286, "localized strongly" to the cell membrane in fed larvae, Figure 8I – see above

As noted in #16 above, we have looked at this TrblLKR/A mutant more carefully and have determined that it does not localize to the cell membrane like SLE/G so this information is removed (update added to Table 1)

REVIEWER #3 found topic interesting but data preliminary.

1- The authors claim changes in the cellular distribution of different proteins and provide examples but changes in intensity in the different cell parts are not quantified. To show their results in a convincing manner changes in the intensity of the different markers between the different parts of the cells (nucleus, cytoplasm, and membrane) should be quantified and statistical analyses should be provided.

We think the approach presented in Fig. 1 and Fig. 2 to measure fluorescence averaged across 90 cell bodies in three independent experiments makes the point clearly – because fat body cells were of different sizes, there were problems aligning the edge of some cells but the presentation shows the change.

In response to starvation, the size of the fat body cell shrinks and the fluorescence of the resulting smaller nucleus as detected with DAPI fluorescence is reduced – similarly the intensity of phalloidin staining of the cell membrane is reduced with starvation. We focus on the levels of Trbl protein and/or its subcellular trafficking and have quantified the change in accumulation of Trbl in Fig. 3 accordingly.

Other data is representative of the influence of UAS-regulated RNAi and transgenes predicted to affect Trbl subcellular localization – because the strength of knockdown or transgene activity is not known, precise measurements of Trbl at the membrane and in the nucleus cannot correlate with a precise measurement of dAkt activity.
We have used markers of nucleus and cell membrane throughout to present our data. For critical data measuring localization during starvation or with the mutants, we present the subcellular distribution in Figs 1 and 2.

2) The immunostainings shown should include scale bars.

Scale bars have been added for all panels

3) Fig 2: markers for nuclei and membrane should be shown, together with HA and Trbl . Quantifications of membrane and nucleus signal in the genetic context analyzed are required.

Markers have been added, quantifications included for key data.

4) Fig 2A shows dAkt over-expressing fat bodies. Trbl protein should be shown in wild type organs. This control is required to compared with the fat bodies where dAkt (2A) and Akt+Trbl (2B) are upregulated.

We add WT Trbl in panels 1A, 2A and 4A where these comparisons are relevant.

5) Fig 2A and B. In the text, the authors mentioned that "Trbl antisera detected accumulation of endogenous Trbl or a Flag-tagged Trbl transgene predominantly in the fat body cell nucleus (respective arrows in 2A and 2B) and weakly in the cytoplasm and at the cell membrane." It would be helpful if the Figs shows labels for both, Trbl or a Flag-tagged Trbl.
In the Fig, it labels only "Trbl". The authors claim it accumulates in the nucleus. A nucleus

© 2025. Published by The Company of Biologists under the terms of the Creative Commons Attribution License (https://creativecommons.org/licenses/by/4.0/).

marker (eg, DAPI) should be used to label the nucleus. The Trbl signal between A and B looks quite different. Why is that? This should be clarified and discussed.

We use nuclear/cell membrane markers now throughout and consistently mark panels

6) The authors stated, "As shown in Fig. 2, Trbl antisera detected accumulation of endogenous Trbl or a Flag- tagged Trbl transgene predominantly in the fat body cell nucleus (respective arrows in 2A and 2B) and weakly in the cytoplasm and at the cell membrane." They however do not mention that that corresponds to tissues over-expressing dAkt. This should be mentioned. Otherwise, it is confusing and difficult to interpret. In the next sentence they claim: "Co- expression of an HA-dAkt showed accumulation of dAkt predominantly at the cell membrane (Fig. 2A' and 2B'; Bischof et al., 2013)." Co-expression with what? Based on the Fig, it seems they meant Trbl but this should be clearly explained in the text.
I find this first part of the results confusing and should be revised to facilitate the understanding by the reader .

We have revised Fig. 3B and C to indicate this

7) The authors claim that r4>HA-myr-dAkt "localized strongly to the fat body cell membrane". Cell membrane markers should be used to mark positively the cell membrane. Besides, it seems HA might be in the membrane, but it is also present in other cytoplasmic regions. To claim that it is enriched in the membrane this signal should be quantified and compared with other cellular structures. The same comment applies to the Trbl signal.
Trbl levels look way stronger in Fig 2C, D than 2A, B. Is the gene expressed at higher levels or protein stability is affected? mRNA and protein quantification should be provided to clarify this important issue.

We use nuclear/cell membrane markers now throughout in this revision.

8) Fig 2E should show the quantification in wild type and Trbl(only)-expressing animals. These controls are required.
The suppression of the myr-dAKt phenotype is the focus here, WT and Trbl eclosion and pupation is 100% so was not presented. We have these controls and can add them if necessary.

9) Fig 2G should be compared with fat bodies where PTEN-RNAi is expressed in the absence of Trbl upregulation. Important control missing.

Pten RNAi and myr-dAkt expression is lethal and co-expression of Trbl is critical to obtain fat bodies for dissection and we make that clear in this revision.

10) Fig 2H is missing Trbl protein in dAkt-RNAi fat bodies with and without Tribl overexpression.

We fix this typo in this version

11) Fig 3A-E. Is that Trbl increase caused by an increase in gene expression? mRNA levels should be quantified and provided.

It is an increase in gene expression documented by the Trbl GFP reporter, which allows us to ask about changes in tissue specific expression – we detail the construction of this Crisper-knockin tool in a revised Materials and Methods.

12) (Fig. 3) The authors claim that "we found increased Trbl protein accumulation at the membrane (arrows, Fig. D and F, respectively) and corresponding reduced levels in the nucleus." The Fig number should be indicated - it is missing. Besides, the nuclear levels, according to the quantification below, are not reduced but increased (see Fig 3F´). I assume the first D should read C´.

We change the text to clarify this.

© 2025. Published by The Company of Biologists under the terms of the Creative Commons Attribution License (https://creativecommons.org/licenses/by/4.0/).

13) (Fig. 4) The authors claim: "Endogenous Trbl and WT Flag-Trbl localized predominantly in the nucleus of fat body cells of well-fed animals (arrows, Fig. 4B and C, respectively), with lower levels in the cytoplasm and levels weakly detectable at the cell membrane." I can´t see that in those panels. Nuclear and membrane markers (phalloidin and DAPI, for example) are required to label those cellular structures.

We add markers in all panels.

14) (Fig. 4) "When stained with Trbl antisera, we detected Flag-TrblFLCR/A and Flag-TrblD/NLK mutations with a distribution similar to WT (arrows mark nucleus, Fig. 4D and 4E respectively)." I disagree. The Trbl pattern in D and E is obviously different.

We add text to emphasize that the accumulation in the nucleus and at cell membrane occurs in all three ("strongly expressed in the nucleus with lower levels in the cytoplasm and at the cell membrane") -to distinguish from SLE/G which is clearly reduced in nucleus and strong at cell membrane.

15) (Fig. 4) "In contrast, the Flag- TrblSLE/G mutation resulted in strong accumulation of Trbl at the cell membrane (arrow, Fig. 4F) with comparatively reduced nuclear and cytoplasmic levels, a distribution profile confirmed by co-localization with phalloidin staining in optical cross-sections (Fig. 4F')." Trbl levels in 4F look higher. Not only in the membrane but all over the cell. Is it a matter of localization or protein stability os also affected. Protein quantification needs to be provided to answer this central question.

We add text "for the mutation Flag-Trbl$^{SLE/G}$, we observed aberrantly high levels of Trbl and Flag staining, with accumulation at the cell membrane with corresponding reduced nuclear and cytoplasmic levels" and reserve quantification for Western analysis.

16) (Fig. 5) Nuclear and membrane markers (phalloidin and DAPI, for example) are required. I can´t see in the examples provided what the authors claim in the text. Signal quantification in the different cellular structures analyzed as well as statistical analysis will be required to show those results in a convincing manner.

We have added these markers and present localization analysis for starvation in Fig 1 and for mutant expression in Fig. 2

17) Fig 6I: the 65 kDa mark in the Trbl-SLE/G is way higher in intensity than the band in the Trbl sample. The authors claim that is 6 times higher. In the western it seems to be way higher. How was that quantification done?

This assertion that levels are 6x higher was based on the analysis of 3 westerns on Chemidoc.

18) (Fig. 6) How is the expression of both transgenes? To claim that the difference in levels is due to protein turnover the authors should show the mRNA levels in both conditions.

The transgenes are inserted at the same landing site - we have compared 2 transgenes of each genotype – the mutants in Fig. 3 are at this landing site, too and show no difference in levels – noting also the effect of SLE/G on the stability of endogenous Trbl, a loci on another chromosome. Thus, we are confident that the effect on stability is due to protein-protein interactions and not changes in RNA levels.

19) (Fig. 7) Nuclei should be labeled and shown in Fig 7F.

This has been done throughout the paper

20) Fig 7H, I: Although the nuclear signal is higher, it is also the background. Those images seem to be captured using different settings. The use of clones could help to show these

© 2025. Published by The Company of Biologists under the terms of the Creative Commons Attribution License (https://creativecommons.org/licenses/by/4.0/).

results in a more convincing manner. Quantifications and statistical analysis of the signal in the different cellular compartments, as in the rest of the MS, will also be required.

We claim that SLE/G inhibition of endogenous Trbl is effectively stabilizing the target, so signal is higher for that result – we have changed the text accordingly – and both levels due to stabilization and subcellular localization – statistical measurement of the fluorescence as carried out for Figs 1 and 2 is not relevant for this synthetic result

---

First decision letter

MS ID#: dev.204493

MS TITLE: The *Drosophila* Tribbles pseudokinase translocates to fat body membrane in response to fasting to modulate insulin sensitivity

Authors: Zachary Fischer, Christopher Nauman, Shima Shayestehpour, Laramie Pence, Samuel Bouyain, Xiaolan Yao and Leonard Dobens

Apologies for taking so long to get back to you, but we were waiting for one last review that did not materialise despite multiple reminders. I am however happy to make a decision based on the two reviews we have received. The referees' comments are appended below, or you can access them online: please go to .

The overall evaluation is positive and we would like to publish a revised manuscript in Development, provided that you are willing to make the minor changes requested by the reviewers. Please provide a point by point response indicating whether and how these changes have been made.

Reviewer 1

SUMMARY OF THE ADVANCE MADE IN THIS PAPER AND ITS POTENTIAL SIGNIFICANCE TO THE FIELD
This study highlights the role of Trbl in regulating insulin signaling, which is crucial for understanding metabolic processes and diseases like diabetes. By elucidating the mechanisms of Trbl's subcellular trafficking and its impact on insulin responses, researchers can better understand how cells adapt to metabolic changes. This knowledge can inform the development of therapeutic strategies for metabolic disorders and enhance our understanding of cellular signaling pathways in development.

SUGGESTIONS TO AUTHORS
The authors have addressed all my concerns. However, before the paper is ready for publication, I suggest revising the format of some figures to enhance reader comprehension. For example, in Fig 1C', the label (Flp>Akti, UAS-lacZ) represents the genotype used, which is common for panels C, C', and C". This should be indicated in the panels, and the specific panel should be labeled with "LacZ," similar to how Fig 1A' is labeled with GFP. Additionally, some figure labels are unreadable, such as in Fig 2D. Other labels, like those in Fig 5C, are extremely small and may be difficult to read in the printed version. In sum, I recommend revising the format of the figures to improve their clarity and enhance the overall quality of the paper.

Reviewer 2

SUMMARY OF THE ADVANCE MADE IN THIS PAPER AND ITS POTENTIAL SIGNIFICANCE TO THE FIELD
The resubmission of the previous work to Development has resulted in an excellent manuscript, full of new findings that suggest Trbl can translocate to fat body plasma membrane, where one of its substrates dAkt is known to transiently associate as a function of insulin signaling. Changes in PIP3 levels (induced via PTEN knockdown) or a constitutive dAKT also change localisation. A very broad range (perhaps the most to date) of Trbl mutations are analyzed in the context of protein

© 2025. Published by The Company of Biologists under the terms of the Creative Commons Attribution License (https://creativecommons.org/licenses/by/4.0/).

localisation (notably nuclear vs membrane and stability, two very important properties related to Trbl downstream functions (confirmed for Slbo/String and Trbl itself). Forced changes in the SLE motif at the beginning of the activation segment (usually a DFG in canonical kinases, always SLE in Trbls), notably Glu to Gly was a dominant negative, sensed downstream through Akt activation and leading to much-increased levels of Trbl; this also occurred via cross-talk with the C-tail. Overall, this interesting piece of work moves the field forwards and is suitable for publication in Development. The finding that Trbl multimerises as a function of key Trbl residues also has interesting implications beyond the fly model system.

SUGGESTIONS TO AUTHORS:
These are minor, the authors have done an excellent job of extending and improving their previous submission. Providing the tracked version of changes was very useful indeed, as is Table 1, especially.

1) Relevant work studying TRIB2 stability in the context of phosphorylation via p70S6k should be cited. (PMID: 24089522)
2) One paper cited (Hill, R., Madureira, P. A., Ferreira, B., Baptista, I., Machado, S., ColaÃ§o, L., Dos Santos, M., Liu, N., Dopazo, A., Ugurel, S. et al. (2017). TRIB2 confers resistance to anti-cancer therapy by activating the serine/threonine protein kinase AKT. Nat Commun 8, 14687) has been retracted, so should be removed.

First revision

Author response to reviewers' comments

Dear Sir or Madam,

We have made the minor changes suggested by reviewers to our manuscript dev.204493 "**The *Drosophila* Tribbles pseudokinase translocates to fat body membrane in response to fasting to modulate insulin sensitivity**" by Fischer et al. We have reformatted the manuscript to comply with your guidelines as well. Changes made are detailed **in bold** below.

Reviewer 1

1. For example, in Fig 1C', the label (Flp>Akti, UAS-lacZ) represents the genotype used, which is common for panels C, C', and C''. This should be indicated in the panels, and the specific panel should be labeled with "LacZ," similar to how Fig 1A' is labeled with GFP.
2. Additionally, some figure labels are unreadable, such as in Fig 2D. Other labels, like those in Fig 5C, are extremely small and may be difficult to read in the printed version. In sum, I recommend revising the format of the figures to improve their clarity and enhance the overall quality of the paper.

We have revised each figure (and supplemental figure) to increase the size font of the labels, and we have carefully ensured that the labels are uniform and clearly visible over the strong fluorescence in some panels throughout (also, we used "b- gal" rather than lacZ).

Reviewer 2

1. Relevant work studying TRIB2 stability in the context of phosphorylation via p70S6k should be cited. (PMID: 24089522)

We thank the reviewer for pointing us to this important and relevant paper (Wang J et al., Impaired phosphorylation and ubiquitination by p70 S6 kinase (p70S6K) and Smad ubiquitination regulatory factor 1 (Smurf1) promote tribbles homolog 2 (TRIB2) stability and carcinogenic property in liver cancer. J Biol Chem 2013; 288:33667-33681) that we have added to the discussion on p12 as follows:

© 2025. Published by The Company of Biologists under the terms of the Creative Commons Attribution License (https://creativecommons.org/licenses/by/4.0/).

"The dependence of Trbl stability on the C-terminal tail recalls the role of specific sequences in the N-terminal region of the Trib2 protein that confer tissue-specific turnover mediated by the combination of p70 S6 kinase phosphorylation and binding of the E3 ligase Smurf1 (Wang et al., 2013b). Thus, the SLE/G mutation may interfere with interactions of a yet to be identified kinase and/or E3 ligase that targets Trbl for turnover."

2. *One paper cited (Hill, R., Madureira, P. A., Ferreira, B., Baptista, I., Machado, S., Colaço, L., Dos Santos, M., Liu, N., Dopazo, A., Ugurel, S. et al. (2017). TRIB2 confers resistance to anti-cancer therapy by activating the serine/threonine protein kinase AKT. Nat Commun 8, 14687) has been retracted, so should be removed.*

We thank the reviewer for alerting us that this paper suggesting that Trib2 is an Akt agonist has been retracted and we have removed it from literature cited. Trib1 has been recently recognized as an Akt agonist, so we have cited this 2023 paper (Singh K, Han C, Fleming JL, *et al*. TRIB1 confers therapeutic resistance in GBM cells by activating the ERK and Akt pathways. *Sci Rep* 2023; **13**:12424). This switch on p11 does not change the discussion materially.

An attached document comparing this revision to the original includes these changes.

Third decision letter

MS ID#: dev.204493R1

MS Title: The Drosophila Tribbles pseudokinase translocates to fat body membrane in response to fasting to modulate insulin sensitivity

Authors: Zachary Fischer; Christopher Nauman; Shima Shayestehpour; Laramie Pence; Samuel Bouyain; Xiaolan Yao; Leonard Dobens

I am happy to tell you that your manuscript has been accepted for publication in Development, pending our standard publication integrity checks.

© 2025. Published by The Company of Biologists under the terms of the Creative Commons Attribution License (https://creativecommons.org/licenses/by/4.0/).
